# CCR5 deficiency accelerates lipopolysaccharide-induced astrogliosis, amyloid-beta deposit and impaired memory function

**DOI:** 10.18632/oncotarget.7453

**Published:** 2016-02-17

**Authors:** Chul Ju Hwang, Mi Hee Park, Jae Yeon Hwang, Ju Hwan Kim, Na Young Yun, Sang Yeon Oh, Ju Kyung Song, Hyun Ok Seo, Yun-Bae Kim, Dae Yeon Hwang, Ki-Wan Oh, Sang-Bae Han, Jin Tae Hong

**Affiliations:** ^1^ College of Pharmacy and Medical Research Center, Chungbuk National University, Cheongju, Republic of Korea; ^2^ College of Veterinary Medicine, Chungbuk National University, Cheongju, Republic of Korea; ^3^ College of Natural Resources and Life Science, Pusan National University, Pusan, Republic of Korea

**Keywords:** Alzheimer's disease (AD), Amyloid beta (Aβ), CC chemokine receptor 2 (CCR2), CC chemokine receptor 5 (CCR5), memory impairment, Pathology Section

## Abstract

Chemokine receptors are implicated in inflammation and immune responses. Neuro-inflammation is associated with activation of astrocyte and amyloid-beta (Aβ) generations that lead to pathogenesis of Alzheimer disease (AD). Previous our study showed that deficiency of CC chemokine receptor 5 (CCR5) results in activation of astrocytes and Aβ deposit, and thus memory dysfunction through increase of CC chemokine receptor 2 (CCR2) expression. CCR5 knockout mice were used as an animal model with memory dysfunction. For the purpose LPS was injected i.p. daily (0.25 mg/kg/day). The memory dysfunctions were much higher in LPS-injected CCR5 knockout mice compared to CCR5 wild type mice as well as non-injected CCR5 knockout mice. Associated with severe memory dysfuction in LPS injected CCR5 knockout mice, LPS injection significant increase expression of inflammatory proteins, astrocyte activation, expressions of β-secretase as well as Aβ deposition in the brain of CCR5 knockout mice as compared with that of CCR5 wild type mice. In CCR5 knockout mice, CCR2 expressions were high and co-localized with GFAP which was significantly elevated by LPS. Expression of monocyte chemoattractant protein-1 (MCP-1) which ligands of CCR2 also increased by LPS injection, and increment of MCP-1 expression is much higher in CCR5 knockout mice. BV-2 cells treated with CCR5 antagonist, D-ala-peptide T-amide (DAPTA) and cultured astrocytes isolated from CCR5 knockout mice treated with LPS (1 μg/ml) and CCR2 antagonist, decreased the NF-ĸB activation and Aβ level. These findings suggest that the deficiency of CCR5 enhances response of LPS, which accelerates to neuro-inflammation and memory impairment.

## INTRODUCTION

Pathological features that appear in the brain of AD patients include deposition of amyloid plaques, neuronal and synaptic loss and activation of astrocytes [1-5]. Activated astrocytes could be implicated in accumulation of amyloid-beta (Aβ), a principal component of senile plaque in Ad brain [6, 7]. Recent data also indicated that astrocytes may act as a source for Aβ because they overexpress β-secretase (BACE1), an enzyme that cleaves amyloid precursor protein (APP) to produce Aβ [8]. These studies suggest that activated astrocytes contribute to the synthesis of Aβ and progress of AD.

Chemokines are produced upon activation by a wide spectrum of inflammatory cell types including astrocytes [9, 10]. Several chemokines, and their receptors and ligands have been found to be upregulated in the AD brain [11]. Monocyte chemoattractant protein 1 (MCP-1), ligand of CC chemokine receptor 2 (CCR2), is found in senile plaque and reactive microglia [12] and promotes activation of astrocytes [13]. It was suggested that neuroinflammation was extended by chemokine-mediated microglial activation and recruitment of astrocytes to the area of neuroinflammation [14, 15]. We previously found that CCR5 deficiency activated astrocytes and Aβ accumulation *via* upregulation of CCR2 [16]. These findings suggest that chemokines, and their receptors and ligands may contribute to the development and/or the progression of AD through modification of astrocyte activation.

It has been continuously reported that brain and systemic LPS injection cause neuroinflammation and thus causing Aβ deposition and memory dysfunction [17, 18]. CCRs expression was induced in the microglia after treatment with LPS [19, 20]. Moreover, CCR5 suppressed LPS-induced microglial neurotoxicity [21] and expression of metalloproteinases (MMPs), important mediators of neuroinflammation in astrocytes [22]. Thus in the present study, we investigated how CCR5 deficiency affects LPS-induced activation of astocytes and its relevance to Aβ accumulation in the neuroinflammatory condition of AD pathogenesis.

## RESULTS

### Accelerated effect of CCR5 knockout on the inflammation-induced memorial impairments in CCR5 mice

**Figure 1:**
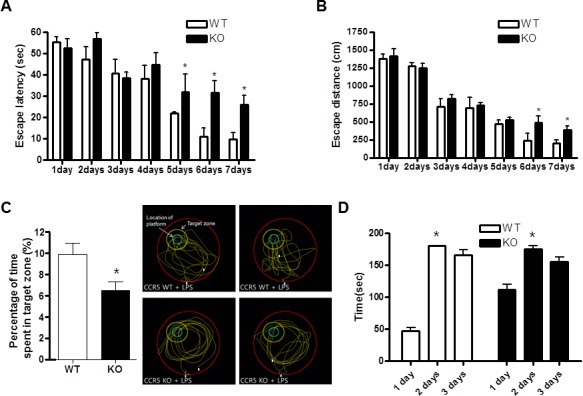
Difference in memory function between CCR5^+/+^ and CCR5^−/−^ mice in water maze test and passive avoidance CCR5^+/+^ and CCR5^−/−^ mice were evaluated for learning and memory of spatial information using the water maze. On days 1-8, each mouse received three training trials in which the mouse was allowed to swim freely about a pool in which a submerged, hidden platform was located. Escape latency, the time required to find the platform **A.** and swimming distance, the distance swam to find the platform **B.** were measured, and after the water maze test, probe trials to measure maintenance of memory were performed **C.** Mice were permitted to swim for a maximum of 60 sec (*n* = 7). A passive avoidance test was performed by step-through methods **D.** Once a mouse enters completely into the dark compartment, it receives an electric shock (0.5 mA, 3 sec) through the stainless steel grid floor. The bar indicates the mean latencies to enter the dark compartment on the learning trial (2 days and 3days) and 24 hr and 48 hr later on the testing trial (1 day). The step-through latency was expressed as mean ± S.E.M (*n* = 7) and the maximum recording time is 300 sec per trial. *Significant difference between CCR5^+/+^ mice (*P* < 0.05).

The water maze test is a widely accepted method of memory testing, and can evaluate spatial learning and memory. Therefore, the Morris water maze was used to determine whether lack of CCR5 influenced spatial learning and memory function. The ability of mice to acquire and recall spatial information was assessed by escape latency in the Morris water maze. The LPS-injected CCR5^+/+^ and LPS-injected CCR5^−/−^ mice exhibited a reduction in escape latency over the training period, but escape latency of LPS-injected CCR5^−/−^ mice was slower than that of LPS-injected CCR5^+/+^ mice (Figure 1A). The escape distance on day 5 to 7 (which may correspond the time to gain completed memory function) was significantly longer in LPS-injected CCR5^−/−^ mice than that of LPS-injected CCR5^+/+^ mice. Swimming distance of LPS-injected CCR5^−/−^ mice was similar to that of LPS-injected CCR5^+/+^ mice until day 4, but was significantly longer than that of LPS-injected CCR5^+/+^ mice after day 5 (Figure 1B). Compared to LPS non injected mice [16], escape latency and distance were much higher after LPS injection in both CCR5^+/+^ and CCR5^−/−^ mice. In our previous study showed that the memory impairment were higher in CCR5^−/−^ mice than CCR5^+/+^ mice [16]. However, there is more severe memory impairment then previous data (LPS non-injection group) when after LPS injection in present study (Supplementary Table 1). These results indicate that the ability of acquisition and recalling of memory was lowered by LPS-injection, and the memory impairment was higher in LPS-injected CCR5^−/−^ mice compared with that of LPS-injected CCR5^+/+^ mice.

After the water maze test, we performed a probe test to investigate maintenance of memory. The time spent in the target area by LPS-injected CCR5^−/−^ mice compared with the LPS-injected CCR5^+/+^ mice during the probe test. Probe test of the LPS-injected CCR5^−/−^ mice group was shorter than that of the LPS-injected CCR5^+/+^ mice group (Figure 1C), suggesting that LPS-injected CCR5^−/−^ mice were more severe in memory maintenance than CCR5^+/+^ mice. Compared to LPS non-injected CCR5^−/−^ mice [16], LPS-injected CCR5^−/−^ mice showed greater memory dysfunction.

To investigate the role of CCR5 in memory function, we compared the memory behavior between CCR5^+/+^ and CCR5^−/−^ mice. Passive avoidance test was performed to test long-term memory function using a step-through protocol. On learning day, animals entered the dark compartment, but the step-through latency of CCR5^−/−^ mice was higher even though learning trials. However, during the testing day, there was no significant difference in time entering the dark compartment (the time maintaining memory function) between CCR5^+/+^ and CCR5^−/−^ mice. The day after testing day, difference of latency was still no significance (Figure 1D). The results of the passive avoidance test could not explain the memory impairment between two groups. However, when we perform the test with LPS injected mice; we found that behavior activity was lower than LPS non injected mice in other study. Also, recent study demonstrated that mice treated with LPS were significantly less active and moved more slowly [23]. So we performed locomotor test for check difference of activity between CCR5^+/+^ and CCR5^−/−^ mice.

To check the difference of activity between CCR5^+/+^ and CCR5^−/−^ mice, we compared the behavior activity by open field test and locomotor activity test between CCR5^+/+^ and CCR5^−/−^ mice. In open field test, the total distance was significantly decreased in CCR5^−/−^ mice after LPS injected (Supplementary Figure 1A). Duration of entering into a central square was also decreased in LPS injected CCR5^−/−^ mice (Supplementary Figure 1B). Additionally, in locomotor activity test which measured with a tilting-type ambulometer was significantly decreased in LPS injected CCR5^−/−^ mice (Supplementary Figure 1C). These data suggest that reason of no difference in passive avoidance test between two groups may be not memory impairment but physical activity by LPS treatment.

### Enhancement effect of CCR5 knockout on the LPS-induced amyloidogenesis in the CCR5 mice brain

**Figure 2:**
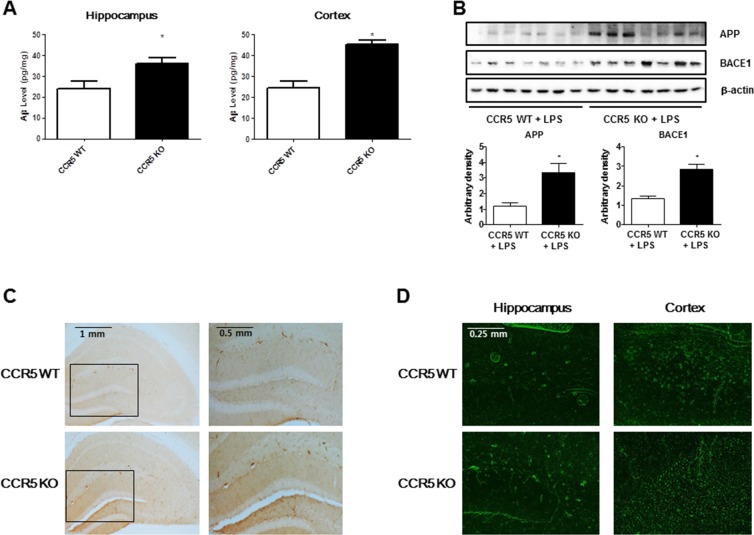
Level of Aβ_1-42_ and amyloidogenesis related proteins (BACE1 and APP) in the brains of CCR5^+/+^ and CCR5^−/−^ mice The amounts of Aβ_1-42_ were assessed by using a specific Aβ_1-42_ ELISA kit as described in the materials and method section **A.** The experiment was performed using hippocampus and cortex regions. Expression of amyloidogenesis related protein (BACE1 and APP) in the brains of CCR5^+/+^ and CCR5^−/−^ mice. The expression of BACE1 and APP was detected by Western blotting using specific antibodies **B.** Immunostaining of Aβ_1-42_ in the brains of CCR5^+/+^ and CCR5^−/−^ mice. 25 μm-thick brain sections were incubated with rabbit polyclonal anti-Aβ_1-42_ antibody **C.** Thioflavin S steining for detection of Aβ accumulation **D.**The distribution of amyloid plaques is shown in the brains of CCR5^−/−^ mice compared with those of CCR5^+/+^ mice. Values measured from each group of mice were calibrated by amount of protein and data are expressed as mean ± S.E.M (*n* = 7). *Significant difference between CCR5^+/+^ mice (*P* < 0.05).

To investigate whether memory impairment in LPS-injected CCR5^−/−^ mice is related with the accumulation of Aβ_1-42_, we measured Aβ_1-42_ level in the brains of LPS-injected CCR5^+/+^ and LPS-injected CCR5^−/−^ mice. The levels of Aβ_1-42_ in LPS-injected CCR5^−/−^ mice were significantly higher than that of LPS-injected CCR5^+/+^ mice in hippocampus and cortex regions (Figure 2A). To investigate whether absence of CCR5 influences amyloidogenesis in the brain, we performed Western blot assay to detect the expression pattern of β-secretase (BACE1) and APP of LPS-injected CCR5^+/+^ and LPS-injected CCR5^−/−^ mice. Expression of BACE1, a key enzyme to generate Aβ_1-42_, was signiticantly higher in the brains of CCR5^−/−^ mice than those of LPS-injected CCR5^+/+^ mice. Expression of APP was also significantly higher in the brains of LPS-injected CCR5^−/−^ mice than those of LPS-injected CCR5^+/+^ mice (Figure 2B). Aβ_1-42_ immunoreactivity had perinuclear localization in the cortex, and had a granular pattern in the hippocampus of LPS-injected CCR5^−/−^ mice. The immunohistochemical analysis by Aβ_1-42_ specific antibody has shown the Aβ deposition in the brain of LPS-injected CCR5^−/−^ mice compared with LPS-injected CCR5^+/+^ mice (Figure 2C). In Thioflavin S staining, Aβ_1-42_ was increased in the brains of LPS-injected CCR5^−/−^ mice compared with LPS-injected CCR5^+/+^ mice (Figure 2D). These results indicate that the increased level of Aβ_1-42_ in the brain and the memory impairment of LPS-injected CCR5^−/−^ mice are closely related. In our previous study showed that the increased levels of Aβ_1-42_ close to 120% higher than CCR5^+/+^ mice in CCR5^−/−^ mice [16]. However, there is much higher increase (about 170%) of Aβ_1-42_ levels in CCR5^−/−^ mice compared to CCR5^+/+^ mice after LPS injection in present study.

Compared to LPS non-injected mice [16], LPS injected mice showed much higher amyloidogenesis as well as neuroinflammation. These evidence suggests that CCR5 knockout accelerated LPS induced amyloidogenesis in CCR5^−/−^ mice brain.

### Enhancement effect of CCR5 knockout on the LPS-induced expression of inflammation related proteins in the CCR5 mice brain

**Figure 3:**
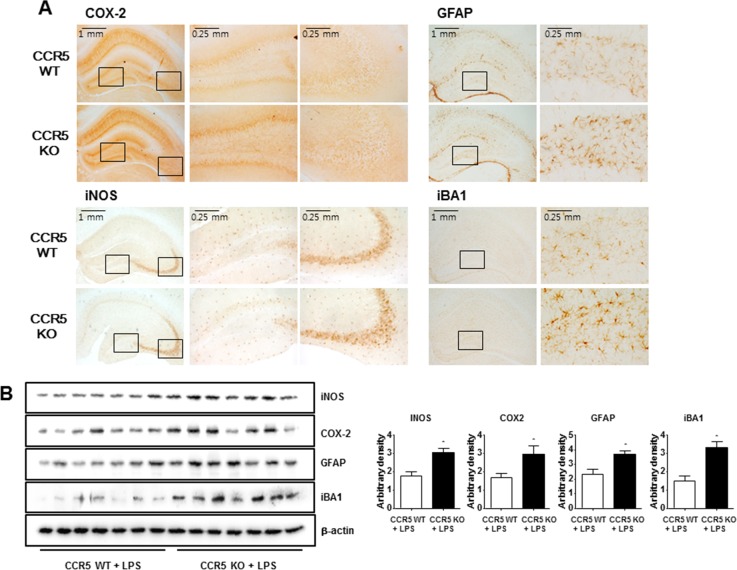
Expression of inflammatory proteins and activation of astrocytes and microglia cells in the brains of CCR5^+/+^ and CCR5^−/−^ mice Brain sections and lysates were incubated with specific antibodies against COX-2, iNOS, GFAP and Iba1 proteins, and then were detected by immunohistochemistry **A.** and Western blot **B.** Data are expressed as mean ± S.E.M (*n* = 7). *Significant difference between CCR5^+/+^ mice (*P* < 0.05).

We previously find that neuroinflammation is critical for Aβ generation, and astrocytes are important contributing factor about neuroinflammation and amyloidogenesis in neuronal cells. We used the methods of Western blot and immunohistochemistry to detect the expression of COX-2 and iNOS in mouse brains. Our data indicate that LPS-injected CCR5^−/−^ mice were significantly increased of these proteins in hippocampus when compared with LPS-injected CCR5^+/+^ mice. Immunostaining for COX-2 and iNOS (Figure 3A) showed significantly higher number of COX-2 and iNOS reactive cells in LPS-injected CCR5^−/−^ mice brain compared to the number in LPS-injected CCR5^+/+^ mice brain.

Since activation of astrocytes and microglia in the brain has been known to be critical in the accumulation of Aβ, brain tissues from LPS-injected CCR5^+/+^ and LPS-injected CCR5^−/−^ mice were stained with glial fibrillary acidic protein (GFAP), a marker protein of astrocytes, to assess astrocytes architecture. GFAP immunohistochemistry revealed a global increase in GFAP immunoreactivity in the brains of LPS-injected CCR5^−/−^ mice as compared with LPS-injected CCR5^+/+^ mice. GFAP-positive cells in the brains of LPS-injected CCR5^−/−^ mice were frequently hypertrophied in comparison to those in LPS-injected CCR5^+/+^ brains (Figure 3A). The numbers of reactive cells of immunostaining for iBA1 (a marker protein of microglia cells) in hippocampus of LPS-injected CCR5^−/−^ mice were also significantly higher compared to the numbers in LPS-injected CCR5^+/+^ (Figure 3A).

Paralleled with the immunostaining data, the results of Western blot also showed that expression of COX-2, iNOS, GFAP and iBA1 were also higher in the brains of LPS-injected CCR5^−/−^ mice compared with LPS-injected CCR5^+/+^ mice detected by Western blot (Figure 3B).

These results suggest that astrocytes were greatly activated in the brains of LPS-injected CCR5^−/−^ mice compared to that of CCR5^+/+^ mice brain.

### Effect of CCR5 knockout on the expression of CCR2 in the brain

**Figure 4:**
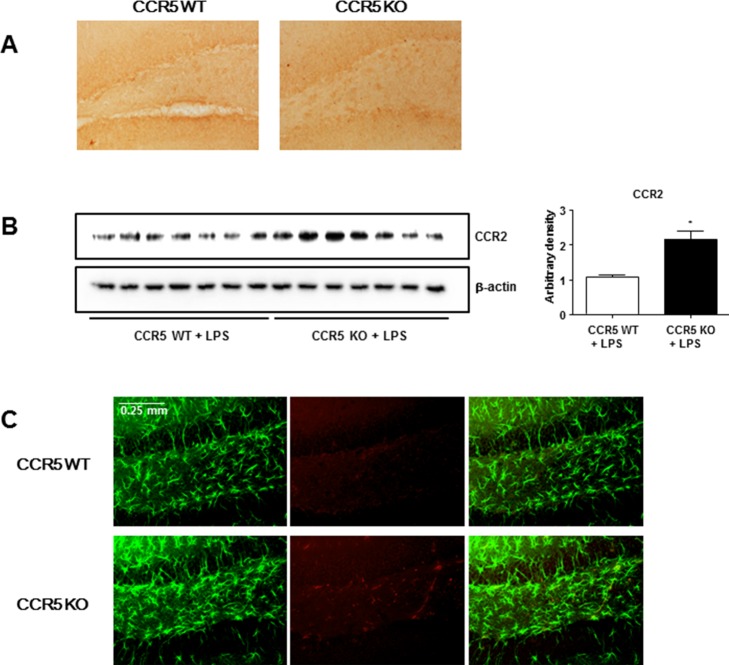
Expression of CCR2 in the brains of CCR5^+/+^ and CCR5^−/−^ mice Brain sections and lysates were incubated with specific antibodies against CCR2 proteins, and then were detected by immunohistochemistry **A.** and Western blot **B.** To evaluate relation of CCR2 and astrocytes activation, double immunofluorescence staining of GFAP with CCR2 **C.** was performed. Brain sections were incubated with specific antibodies against GFAP (green) and CCR2 (red) proteins. Data are expressed as mean ± S.E.M (*n* = 7). *Significant difference between CCR5^+/+^ mice (*P* < 0.05).

Recent study has suggested that absence of CCR5 could induce the activation of CCR2, which leads to the activation of astrocytes. Thus, expression of CCR2 in the brains was performed by Western blot method. Expression of CCR2 in the brains of LPS-injected CCR5^−/−^ mice was higher than that of LPS-injected CCR5^+/+^ mice. Immunohistochemistry of CCR2 in the brains also showed that CCR2 was overexpressed in the neuronal cell membrane of LPS-injected CCR5^−/−^ mice, whereas CCR2 was poorly expressed in the brains of LPS-injected CCR5^+/+^ mice (Figure 4A). Paralleled with the immunostaining data, the results of Western blot also showed that expression of CCR2 was also higher in the brains of LPS-injected CCR5^−/−^ mice compared with LPS-injected CCR5^+/+^ mice detected by Western blot (Figure 4B). To demonstrate that CCR2 could be involved with activation of astrocytes, double immunofluorescence staining of GFAP and CCR2 was performed. Double immunofluorescence staining showed that signifcant higher immunoreactive cells against GFAP was observed in the brains of LPS-injected CCR5^−/−^ mice than those of LPS-injected CCR5^+/+^ mice. In the brains of LPS-injected CCR5^+/+^ mice, CCR2 was normally expressed and a small number of cells were double positive for GFAP and CCR2 (Figure 4C). The results suggest that the absence of CCR5 leads to increased expression of CCR2, which in turn activates astrocytes and Aβ production *via* a compensative mechanism.

### Enhancement effect of CCR5 knockout on the LPS-induced MCP-1 levels in the CCR5 mice brain

**Figure 5:**
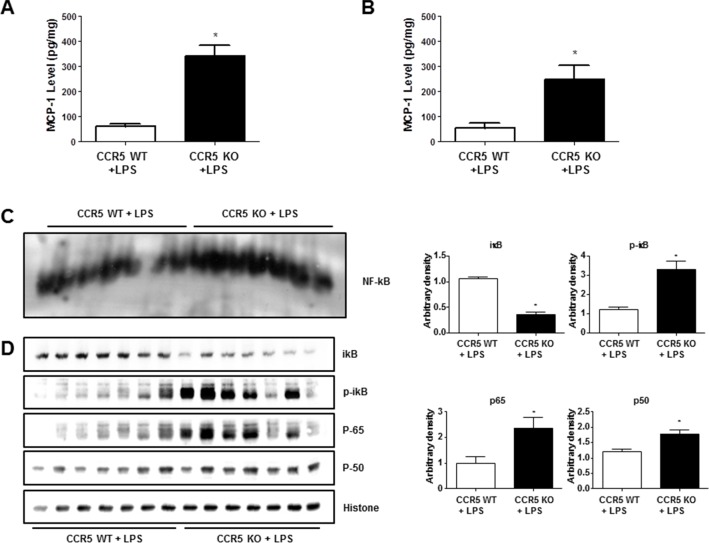
Level of MCP-1 and activation of NF-ĸB in the brains of CCR5^+/+^ and CCR5^−/−^ mice The amounts of MCP-1 were assessed by using a specific MCP-1 ELISA kit as described in the materials and method section. The experiment was performed using hippocampus **A.** and cortex **B.** regions. DNA binding activity of NF-ĸB in CCR5^+/+^ and CCR5^−/−^ mice brain was determined by EMSA **C.** Expression of p50, p65, p-iĸB and total iĸB in CCR5^+/+^ and CCR5^−/−^ mice brain were determined by western blots **D.** Values measured from each group of mice were calibrated by amount of protein and data are expressed as mean ± S.E.M (*n* = 7). *Significant difference between CCR5^+/+^ mice (*P* < 0.05).

MCP-1 is one of the most important for neuroinflammation, NF-ĸB activation as well as amyloidogenesis [8, 24, 25]. Our previous study shows that the absence of CCR5 can lead the increment of CCR2 expression [16]. Also, recent study revealed that chemokines such as MCP-1(CCL2) activate astrocytes *via* CCR2 [13]. To investigate whether neuroinflammation in LPS-injected CCR5^−/−^ mice is related with the expression of MCP-1, we measured MCP-1 level in the brains of LPS-injected CCR5^+/+^ and LPS-injected CCR5^−/−^ mice. LPS injection elevated MCP-1 level compared to non-treated group in both CCR5 wild and knockout mice. However, the levels of MCP-1 in LPS-injected CCR5^−/−^ mice were significantly higher than that of LPS-injected CCR5^+/+^ mice in hippocampus (Figure 5A) and cortex (Figure 5B) regions.

### Effect of CCR5 knockout on the DNA binding activity of NF-ĸB in the brain

The activation of NF-ĸB plays a critical role in the neuro-inflammation since it control several gens involving neuroinflammation as well as amyloidogenesis. To determine whether absence of CCR5 could increase the activation of NF-ĸB after LPS injection, we measured the DNA binding activity of NF-ĸB by EMSA, and translocations of p50 and p65 into nucleus and the protein expression were determined by Western blotting. Much higher DNA binding activity of NF-ĸB (Figure 5C) and the nuclear translocations of p50 and p65 (Figure 5D) were observed in the brain of CCR5^−/−^ compared to CCR5^+/+^ mice when after LPS injection.

### Involvement of CCR2 on activation of NF-ĸB, Aβ accumulation and MCP-1 expression in microglia cells and cultured astrocytes isolated from CCR5^−/−^ mice

**Figure 6:**
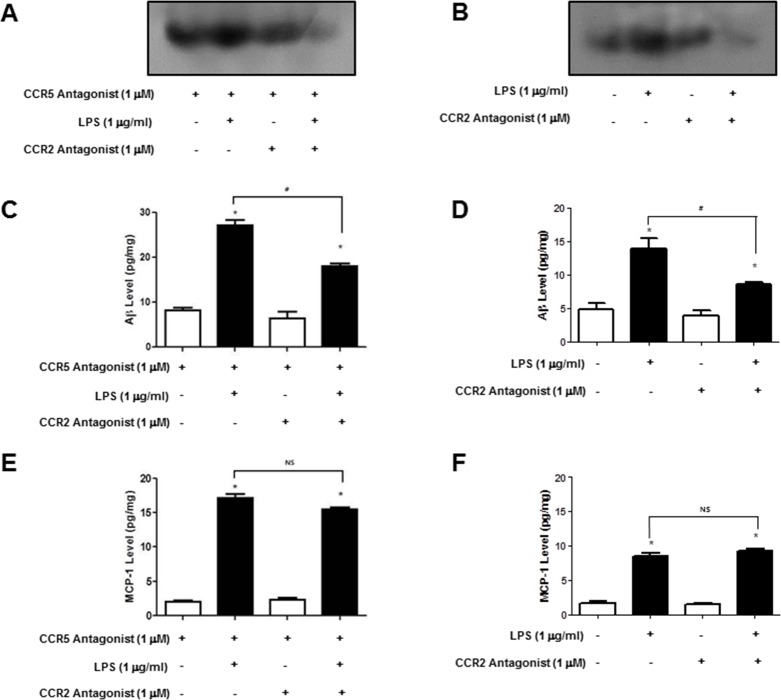
Activation of NF-ĸB and level of Aβ1-42 and MCP-1 in DAPTA treated BV-2 cells and cultured astrocytes isolated from CCR5^−/−^ mice after CCR2 antagonist LPS-induced Primary cultured astrocytes (isolated from CCR5^−/−^ mice) and DAPTA treated microglial BV-2 cells were treated with CCR2 antagonist, and then activation of NF-ĸB was determined by EMSA **A.** and **B.** level of Aβ_1-42_ was measured by using a specific Aβ_1-42_ ELISA kit **C.** and **D.** MCP-1 level was measured by using a specific ELISA kit **E.** and **F.** Data are expressed as mean ± S.E.M (*n* = 7). *Significant difference against from LPS-non treated group (*P* < 0.05) and ^#^Significant difference between LPS treated groups (*P* < 0.05).

To further demonstrate the involvement of CCR2 in CCR5-mediated amyloidogenesis NF-ĸB activation, Aβ accumulation and MCP-1 level were determined in astrocytes and microglia cells. We used BV-2 cells treated with CCR5 antagonist, D-ala-peptide T-amide (DAPTA) and cultured astrocytes isolated from CCR5^−/−^ mice treated with LPS (1μg/ml) and CCR2 antagonist (Santa Cruz Biotechn, Santa Cruz, CA, USA), and measured the DNA binding activity of NF-ĸB by EMSA. Aβ accumulation was also determined by Aβ specific ELISA kit. The treatment of CCR2 antagonist, decreased the DNA binding activity of NF-ĸB in DAPTA treated BV-2 cells (Figure 6A) and cultured astrocytes isolated from CCR5^−/−^ mice (Figure 6B). Aβ levels were also decreased after CCR2 antagonist treatment in DAPTA treated BV-2 cells (Figure 6C) and cultured astrocytes isolated from CCR5^−/−^ mice (Figure 6D). Additionally, the level of MCP-1 protein was also determined by specific ELISA kit. However, MCP-1 levels were not changed by treatment of CCR2 antagonist in DAPTA treated BV-2 cells (Figure 6E) and cultured astrocytes isolated from CCR5^−/−^ mice (Figure 6F). These data show that treatment of CCR2 antagonist could decrease NF-ĸB activity and amyloidogenesis through blocking to bind CCR2 and its ligand MCP-1.

### Effect of CCR5 knockout on cell death in CCR5 mice brain

Activation of astrocytes could influence Aβ accumulation which causes apoptotic neuronal cell death. To investigate apoptosis of neuronal cells in the brain, we performed TUNEL assay in brain sections. In the brains of LPS-injected CCR5^−/−^ mice, TUNEL-positive apoptotic cells were frequently observed and the indication of apoptosis was significantly higher in LPS-injected CCR5^−/−^ mice than that of CCR5^+/+^ mice (Supplementary Figure 2).

## DISCUSSION

In the present study, it was shown that the deficiency of CCR5 in LPS treated CCR5^−/−^ mice resulted in the dysfunction of memory capacity. Associated with the memory dysfunction, the amount of Aβ_1-42_ and expression of amyloidogenesis related proteins (BACE1 and APP) were much higher in the brains of LPS treated CCR5^−/−^ mice than those of LPS treated CCR5^+/+^ mice. These results are similar, but higher extent to previous findings in LPS untreated CCR mice [16], and indicate again that the deficiency CCR5 in the LPS treated CCR5^−/−^ mice leads to increased Aβ_1-42_ accumulation, and thus impaired memory function.

Neuroinflammation could activate astrocytes in the brain of AD patients which gradually accumulate Aβ_1-42_, and the amount of Aβ_1-42_ correlates positively with the extent of AD pathology [26]. We and other scientists have demonstrated that neuroinflammation induced by LPS activated astrocytes, and thus activated astrocytes generate Aβ in the brain [16, 25, 27, 28]. Aβ accumulation in the major human cell types associated with senile plaques revealed that astrocytes generate higher levels of Aβ [29, 30]. Moreover, recent studies have also suggested that astrocytes could act as a source for Aβ because they overexpress BACE1 in response to chronic stress [8]. Our data showed that inflammation much great neuroinflamation, activation of astrocytes and Aβ accumulation were found in LPS-injected CCR5^−/−^ mice compared to those in CCR5^+/+^ mice. Previously we also showed CCR5^−/−^ mice activated astrocytes and induced Aβ accumulation in the mice brain even without treatment of LPS [16]. Thus, these data confirmatively demonstrated that absence of CCR5 accelerates amyloidogenesis and thus, memory impairment.

In present study showed that not only phospholylation of IĸB but also translocation of p50 and p65 protein in LPS-injected CCR5^−/−^ mice. Binding capability with NF-ĸB was also higher in LPS-injected CCR5^−/−^ mice. NF-ĸB regulates pro-inflammatory cytokine expressions such as IL-6, IL-1β and TNF-α, which are present at increased levels in the brains of AD patients [31, 32]. These cytokines (IL-6 and IL-1β) increase not only inflammation, but also apoptotic cell death in the brain [33]. Injections of IL-1α or IL-1β in the brain also exhibited increased AD associated with plaque formation [34]. Also, the NF-κB controls the expression of APP and BACE1, which enhances Aβ formation [35]. According to these evidences, LPS injection can induce neuroinflammation and amyloidogenesis through NF-ĸB pathway in brain. The level of MCP-1 is upregulated in the brain during several neurodegenerative and acute diseases. MCP-1 gene is regulated by NF-ĸB phospholyration [36]. In primary cultured astrocytes from rat, NF-ĸB pathways are important for the release of proinflammatory cytokines such as IL-1β and TNF-α which can induce CCL2-stimulation [37]. Recently, it was demonstrated that LPS-injection enhanced NF-ĸB dependent MCP-1 gene expression in rat [38]. Neuroinflammation was also decreased after LPS injection in MCP-1^−/−^ mice [39]. In our present data showed that NF-ĸB activity as well as NF-ĸB DNA-binding capacity is higher in CCR5^−/−^ mice compared to CCR5^+/+^ mice when after LPS injection. According to high activity of NF-ĸB, levels of MCP-1 was also higher in LPS-injected CCR5^−/−^ mice than that of CCR5^+/+^ mice. Additionaly, MCP-1 which is ligand of CCR2 was also higher in CCR5^−/−^ mice compared to CCR5^+/+^ mice after LPS injection. In our previously study [16], CCR2 expression was increased close to 130% in CCR5^−/−^ mice brain compared to CCR5^+/+^ mice brain without LPS injection. However, in present study, there are about 200% increase in CCR5^−/−^ mice brain compared to CCR5^+/+^ mice brain after LPS injection. Also, increase range of Aβ accumulation (LPS non injection: 120%, LPS injection: 170%) and neuronal cell death (LPS non injection: 120%, LPS injection: 300%) between CCR5^+/+^ mice and CCR5^−/−^ mice were significantly expand after LPS injection. These data suggest that enhancement of activation of astrocytes may be critically implicated in the accumulation of Aβ and memory dysfunction in the CCR5^−/−^ mice, and it was amplified by the treatment with LPS through NF-ĸB pathway.

We previously also found that the extensive expression of CCR2 in the brains of CCR5^−/−^ mice was higher than that of CCR5^+/+^ mice. Among the CC receptors, CCR2 shares one or more ligands with CCR5 [40-42], and the increased expression of CCR2 is the result of a compensating response for the absence of CCR5 [43]. A recent study revealed that chemokines such as MCP-1(CCL2) activate astrocytes *via* CCR2 [13]. Thus, it is possible that the increased expression of CCR2 by compensation with CCR5 in CCR5^−/−^ mice may cause astrocyte activation, which leads to deposition of Aβ causing memory dysfunction [8, 25, 30]. Thus, change of specific chemokines could be significant in pathphysiological alteration in CCR5^−/−^ mice. There are five known members of the monocyte chemoattractant protein (MCP) family, designated as CCL2, CCL8, CCL7, CCL13, and CCL12 (MCP-1-5, respectively). CCL2 is the most potent at activating signal transduction pathways leading to monocyte transmigration [44]. In the CNS, CCR2 expression has been reported on various cell types, including neurons, astrocytes, microglia, neural progenitor cells [45-49]. During normal conditions, expression of CCR2 seems to be at consistently low levels. CCR2 expression in astrocytes and microglia seems to be quite heterogeneous and subject to significant upregulation during an inflammatory response [50-52]. Indeed, behavioral studies have shown that mice lacking CCR2 display a macrophage recruitment in several injury and disease models [53]. Following injury to the brain, astrocytes produce CCL2 mRNA within 3 h, before the accumulation of inflammatory mononuclear cells [10]. Interestingly, other studies have shown that CCL2-signalling can affect Aβ pathology in animal models of AD [24, 54]. In APP expressing transgenic mouse model, increased Aβ pathology accelerated expressing of CCL2 [54]. Additionally CCL2 and CCR2 expression are well reported in the many studies in conditions of nerve injury [55-58] and tissue inflammation [59]. Recently studies of human indicate that CCL2 levels are increased in the brains of AD patients, and CCL2 co-localizes with senile plaques [60, 61]. Other study suggests that the changement of CCL2-levels in MCI patients reflect this immunological activity [62]. Recently study reports that MCP-1 levels were significantly increased in serum and brain, when 6hrs after LPS injection [39]. These studies support that CCL2 associated inflammation and microgliosis might have detrimental effects on Aβ metabolism. Therefore, lack of CCR5 caused CCR2 expression as a mean of conversely compensation mechanism leading to more amyloidogenesis and more severe impairment in CCR5^−/−^ mice with LPS treatment.

CCR5 deficiency-mediated memory dysfunction could be related with the neuroprotective role of CCR5. Several CCR members are expressed in the CNS cell types including astrocytes and microglia [45], and their ligands such as MIP-1β have been found to be highly expressed in cerebrospinal fluid (CSF). It was also noteworthy that CCR5 antagonist treated neurons and glial cells cultured from rat showed less toxicity against Aβ as compared with cells cultured from control wild mice [63]. However, the chemokine receptor CCR5 is not a necessary inflammatory mediator in brain injury. After hippocampal injury, not only expression but also mRNA level of CCR2 and CCR3 were increased in CCR5 deficient mice compared to CCR5 wild type mice [43]. Expression of CCR2 was different between wild type and mutant type of CCR5 mice brains, suggesting CCR2 may be affected by CCR5, and these changes could be amplified by LPS leading greater of memory dysfunction. LPS produced significantly higher levels of cytokines and chemokines, in brain for IL-1a, IL-6, MCP-1, MIP-1a, and TNF and in serum for IL-6 [64]. Because of the high level of CCR2 expression, deficiency in CCR5^−/−^ mice may not ensure the neuroprotective role of chemokines leading to loss the maintenance of memory function.

In addition, neuronal cell differentiation could be mediated by CCR5. Neuronal differentiation is an important factor to prevent neurodegeneration in Alzheimer's disease and related disorders [65]. In our previous study show that neuronal cell differentiation was abolished by CCR5 disruption [66].

In this study, it is notable that the cell death in the brains of CCR5^−/−^ mice was significantly higher than in the brains of CCR5^+/+^ mice. Thus, it is also possible that the lack of CCR5 may not be sufficient to prevent neuronal cell death from elevated Aβ deposit. Taken together, these findings suggest that absence of CCR5 leads to accelerate LPS-induced astrocytes activation, and Aβ deposition through an increase in CCR2, and can possibly result in more severe impaired memory function.

## MATERIALS AND METHODS

### LPS induced memory impairment mouse model

The age (4∼5 month old), sex (male) and weight (26-27 g) matched CCR5 wild type (CCR5^+/+^) mice and CCR5 knock out (CCR5^−/−^) mice were maintained in accordance with the guidance of the National Institute of Toxicological Research for the care and use of laboratory animals. All of the experimental procedures were approved by the Animal Care and Use Committee (IACUC) of Chungbuk National University (approval number: CBNUA-144-1001-01). The CCR5^+/+^ and CCR5^−/−^ mice were purchased from The Jackson Laboratory (Bar Harbor, Maine 04609). CCR5^−/−^ mice have no overt developmental abnormalities. Control mice (B6129PF2/J) were F2 hybrid mice from the C57BL/6J-AW-J and 129P3/J parental strains. CCR5^−/−^ mice (B6;129P2-Ccr5tm1Kuz/J) have the entire coding region of the CCR5 gene deleted from the parental strains, B6;129P2-Ccr5tm1kuz and B6;129P2-Cmkbr5tm1Kuz. The LPS (Sigma, St. Louis, MO; final concentration of 0.1 mg/ml) was dissolved, and aliquots in saline were stored at − 20°C until use. The ip injection (0.25 mg/kg) of LPS or vehicle (saline) was administered daily for 7 days.

### Water maze test

Water maze test was described by Morris et al. (1984) using the SMART-CS program and equipment (Panlab, Barcelona, Spain). A circular pool (Height: 35 cm, Diameter: 100 cm) was filled with milky water and maintained at 22∼25°C. An escape platform (Height: 14.5 cm, Diameter: 4.5 cm) was then submerged 0.5∼1 cm below the surface of the water in the northeastern quadrant position of the pool. During testing, mice were placed in the plastic pool, allowed 60 sec to find the hidden platform and remain on the platform for 10 sec. Mice that did not find the platform within 60 sec were placed on the platform for 10 sec at the end of the trial. Escape latency (the time required to find the platform), escape distance (the distance swam to find the platform), swimming speed, and swimming patterns were monitored for 8 days using a camera positioned above the counter of the pool. The camera was connected to a computer running the SMART-LD program (Panlab).

### Probe test

To assess memory consolidation, a probe test was performed 48 hr after the water maze test. For the probe test, the platform was removed from the pool and the mice were allowed to swim freely. The swimming pattern of each mouse was monitored and recorded for 60 s using the SMART-LD program (Panlab). Consolidated spatial memory was estimated by the time spent in the target area which adjacent to platform.

### Passive avoidance test (Step-through test)

The passive avoidance test is a widely accepted simple and rapid means of memory testing. Passive avoidance response was determined using a “step-through” apparatus (Med Associated, St. Albans, VT, USA), which consisted of an illuminated and a dark compartment (each 20.3 × 15.9 × 21.3 cm) adjoining each other through a guillotine door. Floors were constructed of 3.175 mm stainless steel rods set 8 mm apart. The test was conducted on 2 consecutive days at the same time of day. On the first day (learning trials), each mouse was placed in the illuminated compartment facing away from the dark compartment. Once the mouse enters completely into the dark compartment, it receives an electric shock (0.5 mA, 3 sec) through the stainless steel grid floor. The time required until the mouse entered into the dark compartment was recorded automatically and described as the step-through latency. On the second day (testing day), the same test procedure was followed. When the mouse did not enter the dark compartment within 300 sec, the test was terminated and a latency of 300 sec was recorded.

### Open field test

Two days after the passive avoidance test, locomotor activity was evaluated by placing mice into an open-field arena. Each mice were individually placed in the center of the open field (50 × 50 × 30 cm) and left to explore freely for 10 min in standard room-lighting conditions. Activity in the plexiglas was quantitated by a computer-operated system [SMART-LD program (Panlab)]. The total distance moved in the arena in 10 min was recorded as a measure of locomotor distance. Frequency and duration of entering into a central square (25 cm × 25 cm) of the open field during the 10 min were automatically recorded and were used as measures of anxiety-related behavior.

### Locomotor activity

Spontaneous locomotor activity was measured automatically with a tilting-type ambulometer (AMB-10, O'Hara, Japan). Each mouse was placed in the activity cage (20 cm in diameter and 18 cm in height) and after an adaptation period of 10 min.

### Western blotting

Brain and spinal cord tissues were homogenized with protein extraction solution (PRO-PREPTM, Intron Biotechnology, Seoul, Korea), and lysed by 60 min incubation on ice. The lysate centrifuged at 15,000 rpm for 30 min at 4°C. Equal amount of protein (40 μg) were separated on a SDS/10%-olyacrylamide gel, and then transferred to a polyvinylidene difluroride (PVDF) membrane (GE Water & Process technologies, Trevose, PA, USA). Blots were blocked for 1 hr at room temperature with 5% (w/v) non-fat dried milk in Tris-Buffered Saline Tween-20 [TBST: 10 mM Tris (pH8.0) and 150 mM NaCl solution containing 0.05% tween-20]. After a short wash in TBST, the membrane was incubated at room temperature with specific antibodies. Rabbit polyclonal antibodies against APP (1:1000, Affinity BioReagents, Golden, CO, USA) and BACE1 (1:500, Sigma, St Louis, MO, USA), and gout polyclonal antibody against CCR2 (1:1000, Abcam, Cambrige, MA, USA) were used in study. The blot was then incubated with the corresponding conjugated anti-rabbit, anti-mouse and anti-gout immunoglobulin G-horseradish peroxidase (1:2000, Santa Cruz Biotechn, Santa Cruz, CA, USA). Immunoreactive proteins were detected with the BM chemiluminescence blotting substrate (Roche Applied Science, Mannheim, Germany).

### Immunohistochemistry

Mice were anesthetized with ether. While under general anesthesia, the mice received intracardiac perfusion with 20 ml of saline, followed by 50 ml of phosphate-buffered saline (PBS) containing 4% paraformaldehyde. After perfusion, the brain was dissected and post-fixed for 2-4 hr in the same fixative, and were then cryoprotected overnight in 30% sucrose prepared in PBS. Serial coronal sections of brain (40 μm) were cut with a freezing microtome.

Sections were rinsed in 0.1M phosphate buffer and treated with 3% hydrogen peroxide for 30 min. After washing in PBS, the sections were incubated overnight at 4°C with antibodies specific for Aβ_1-42_ (1:2000, Covance, Berkely, CA) or GFAP (1:5000, Abcam), incubated in biotinylated goat anti-rabbit IgG (1:2000, Vector Laboratories, Burlingame, CA, USA) for 1 hr at room temperature (RT), followed by incubation in avidin-conjugated peroxidase complex (ABC, 1:200, Vector Laboratories) for 30 min at RT, and the peroxidase reaction was visualized using 3, 3`-diaminobenzidine tetrahydrochloride (DAB, 0.02%) as the chromogen. The sections were rinsed, mounted on poly-glycine-coated slides, dehydrated, and cover-slipped for light microscopy and photography.

### Immunofluorescence

Sections were treated with 10% bovine serum albumin in PBS for 1 hr at RT, incubated overnight at 4°C with CCR2 (1:300, Goat monoclonal, R&D Systems, Minneapolis, MN, USA) or GFAP (1:300, mouse, Santa Cruz Biotechn, Santa Cruz, CA, USA), followed by incubation in anti-mouse IgG conjugated with Alexa 488 (1:300 dilution, Molecular Probes, Eugene, OR, USA) or anti-goat IgG conjugated with Alexa 568 (1:300 dilution, Molecular Probes) for 40 min at RT. Finally, the sections were rinsed, mounted on slides, and cover-slipped for fluorescence microscopy and photography using ApoTome microscopy (Carl Zeiss, Thornwood, NY, USA).

### Measurement of Aβ_1-42_

Lysates of brain tissue were obtained through protein extraction buffer containing protease inhibitor. Aβ_1-42_ levels were determined using a specific ELISA Kit (Immuno-Biological Laboratories Co., Ltd., Takasaki-Shi, Gunma, Japan). In brief, 100 μl of sample was added into the precoated plate and was incubated overnight at 4°C. After washing each well of the precoated plate with washing buffer, 100 μl of labeled antibody solution was added and the mixture was incubated for 1 hr at 4°C in the dark. After washing, chromogen was added and the mixture was incubated for 30 min at room temperature in the dark. Finally, the resulting color was assayed at 450 nm using a microplate absorbance reader (SunriseTM, TECAN, Switzerland) after adding stop solution.

### Measurement of MCP-1

Lysates of brain tissue were obtained through protein extraction buffer containing protease inhibitor. MCP-1 levels were determined using a specific ELISA Kit (R&D Systems, Minneapolis, MN, USA). In brief, 100 μl of sample was added into the precoated plate and was incubated 3hr at room temperature. After washing each well of the precoated plate with washing buffer, 100 μl of labeled antibody solution was added and the mixture was incubated for 1 hr at room temperature. After washing, chromogen was added and the mixture was incubated for 30 min at room temperature in the dark. Finally, the resulting color was assayed at 450 nm using a microplate absorbance reader (SunriseTM, TECAN, Switzerland) after adding stop solution.

### Detection of cell death

In order to investigate apoptotic cell death, immunohistochemistry for terminal deoxynucleotidyl transferase-mediated dUTP nick end-labeling (TUNEL) assay for specific apoptosis markers were performed. Alternatively, sections were submitted to the TUNEL assay at various time points to determine DNA fragmentation according to manufacturer's instructions (In Situ Cell Death Detection kit, POD, Roche). In brief, tissues were post-fixated by 4% paraformaldehyde for 30 min, and then washed by PBS. Tissues were incubated with permeabilization solution (0.1% Triton X-100 and 0.1% sodium citrate in PBS) for 2 min on ice. The sections were then incubated in the mixture of labeling solution (450 μl) and enzyme solution (50 μl) for 1 hr at 37°C and washed 3 times on 0.1 M PBS for 5 min each according to manufacturer's instructions. Next, the sections were incubated with DAPI for 15 min at 37°C. Finally, the sections were rinsed, mounted on slides, and cover-slipped for fluorescence microscopy and photography using ApoTome microscopy (Carl Zeiss).

### Gel electromobility shift assay (EMSA)

Gel shift assays were performed according to the manufacturer's recommendations (Promega, Madison, WI). Briefly, 5 × 106 cells was washed twice with 1× PBS, followed by the addition of 1 ml of PBS, and the cells were scraped into a cold Eppendorf tube. Cells were spun down at 13,000 rpm for 5 min, and the resulting supernatant was removed. Cell were suspended in 400 μl of solution A containing 10 mM HEPES, pH 7.9, 1.5 mM MgCl2, 10 mM KCl, 0.5 mM dithiothreitol, 0.2 mM phenylmethylsulfonyl fluoride; vigorously vortexed; allowed to incubate on ice for 10 min; and centrifuged at 12,000 rpm for 6 min. The pelleted nuclei were resuspended in solution C (solution A + 420 mM NaCl, 20% glycerol) and allowed to incubate on ice for 20 min. The cells were centrifuged at 15,000 rpm for 15 min, and the resulting nuclear extract supernatant was collected in a chilled Eppendorf tube. Consensus oligonucleotides were end-labeled using T4 polynucleotide kinase and [γ-32P] ATP for 10 min at 37°C. Gel shift reactions were assembled and allowed to incubate at room temperature for 10 min followed by the addition of 1 μl (50,000-200,000 cpm) of 32P end-labeled oligonucleotide and another 20 min of incubation at room temperature. Subsequently 1 μl of gel loading buffer was added to each reaction and loaded onto a 6% nondenaturing gel and electrophoresis until the dye was four-fifths of the way down the gel. The gel was dried at 80°C for 1 hr and exposed to film overnight at −70°C.

### Astrocytes cells culture

Astrocytes were prepared from the cerebral cortex of 1-day-old neonatal rat. The cerebral cortex was dissociated into a single-cell suspension by trypsinization and mechanical disruption. The cells were seeded on PLL (0.1 mg/ml, Sigma) coated culture flasks and incubated in Dulbecco's modified eagle medium (DMEM)/F-12 (Invitrogen, Carlsbad, CA) containing 5% fetal bovine serum (FBS) (Invitrogen). The culture medium was replaced at 24 h and every 3 days thereafter. After 10-12 days, the cultures became confluent and loosely attached microglia and oligodendrocyte precursor cells were removed from the cell monolayer. Astrocytes were subsequently detached using trypsin-EDTA and plated into PLL-coated 6-well plates.

### Statistics

Data in Figure 1B and 1C were analyzed by two-way ANOVA, and all other data were analyzed using ANOVA followed by Dunnett's *post hoc* tests using GraphPad Prism 4 software (Version 4.03, GraphPad software, Inc.).. The level of significance was set at *P* < 0.05.

## SUPPLEMENTARY FIGURES AND TABLES


