# Supplementary material for: CCR5 deficiency accelerates lipopolysaccharide-induced astrogliosis, amyloid-beta deposit and impaired memory function
Source: Oncotarget. 2016 Feb 17;7(11):11984–99. doi: 10.18632/oncotarget.7453 (PMC4914263; doi:10.18632/oncotarget.7453)
Supplement: Supplementary file 1 [file oncotarget-07-11984-s001.pdf]

Reciprocal crosstalk between tumour and brain parenchyma instructs histopathological features in glioblastoma

Suppl. Fig. 1. MRI anatomical images for a P13 angiogenic tumour in mouse brain.

Suppl. Fig. 2. Phenotypes of glioma stem-like cell-derived xenografts.

Suppl. Fig. 3. Gating strategy for sorting and multicolor phenotypic analysis.

Suppl. Fig. 4. In vitro spheroid cultures.

Suppl. Fig. 5. Neuropathological analysis of Angpt2^{positive} vessels.

Suppl. Fig. 6. Selected networks for putative protein-to-protein interactions between tumor cells and ECs.

Suppl. Table 1. Clinical patient data and chromosomal aberrations of corresponding human glioblastoma biopsies.

Suppl. Table 2. Xenografts derived in NOD/SCID mice used in the study.

Suppl. Table 3. List of antibodies.

Suppl. Table 4. Comparison of differentially expressed genes (DEGs) between angiogenic (P13), intermediate (P3) and invasive (P8) tumour cells.

Suppl. Table 5. DAVID analysis for differentially expressed genes (DEGs) between angiogenic (P13) and invasive (P8) tumour cells.

Suppl. Table 6. Upstream regulator analysis between angiogenic (P13) and invasive (P8) tumour cells.

Suppl. Table 7. DAVID analysis for differentially expressed genes (DEGs) between endothelial cells of the angiogenic tumour (P13) and normal brain.

Suppl. Table 8. Comparison of differentially expressed genes (DEGs) between endothelial cells (ECs) of angiogenic and intermediate tumour versus normal mouse brain ECs.

Suppl. Table 9. Upstream regulator analysis between endothelial cells of the angiogenic tumour (P13) and normal brain.

Suppl. Table 10. Comparison of differentially expressed genes in tumour and ECs.

Suppl. Table 11. Integrative analysis of protein-protein interactions between tumour and stromal ECs.

Suppl. Table 12. Comparison with study by Dieterich et al.

SUPPLEMENTARY MATERIAL AND METHODS

Array comparative genomic hybridization (aCGH)

Each patient GBM was analyzed for copy number aberrations using SurePrint G3 Human 2x400k CGH microarrays (Agilent Technologies) as previously described (1). Briefly, genomic DNA was extracted using the DNAeasy Blood and Tissue Kit (Qiagen). DNA was fragmented (200-500bp) by DNAse1 (rDNAse1, Ambion) and labeled with the BioPrime aCGH Genomic labeling Kit (Invitrogen) with Cy3 and Cy5 dyes (GE Healthcare) following standard Agilent protocols. Female DNA pool (Promega) was used as a reference. The slides were scanned at 3µm resolution (Agilent High-Resolution Microarray scanner), the image data was extracted using Feature Extraction (Agilent Technologies). Aberrations were called using the ADM2 algorithm with a threshold setting of 25, centralization ‘on’ and an aberration filter with a minimal number of probes=5 and a minimal AvgAbsLogRatio=0.45.

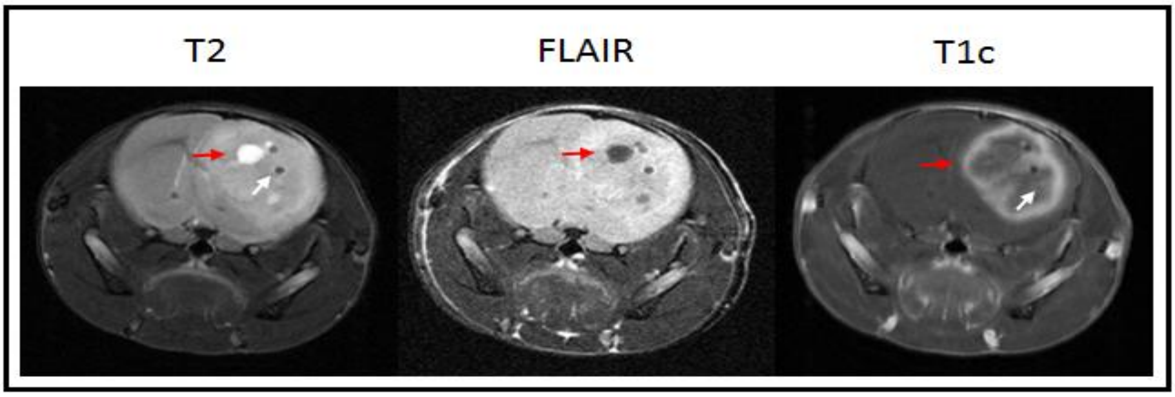

Suppl. Fig 1. MRI anatomical images for a P13 angiogenic tumour in mouse brain (Related to **Fig. 1C**) T2-weighted MRI (left panel) shows a large heterogenous tumour developed in the right hemisphere expanding to the left hemisphere. Dark spots (white arrow) correspond to angiogenic blood vessels and white spots (red arrow) reflect fluid accumulation. The T2-Fluid Attenuated Inversion Recovery (FLAIR) MRI image (center panel) suggests the central white spot in the T2-weighted serie (red arrow) to contain CSF. T1-weighted MRI (right panel) after injection of Gd-based contrast agent reveals a ring of contrast enhancement (red arrow), associated with leaky vasculature, surrounding a central necrotic region (white arrow), in a pattern similar to those observed in clinical Glioblastomas. FLAIR: Fluid Attenuated Inversion Recovery, CSF: CerebroSpinal Fluid, Gd: Gadolinium

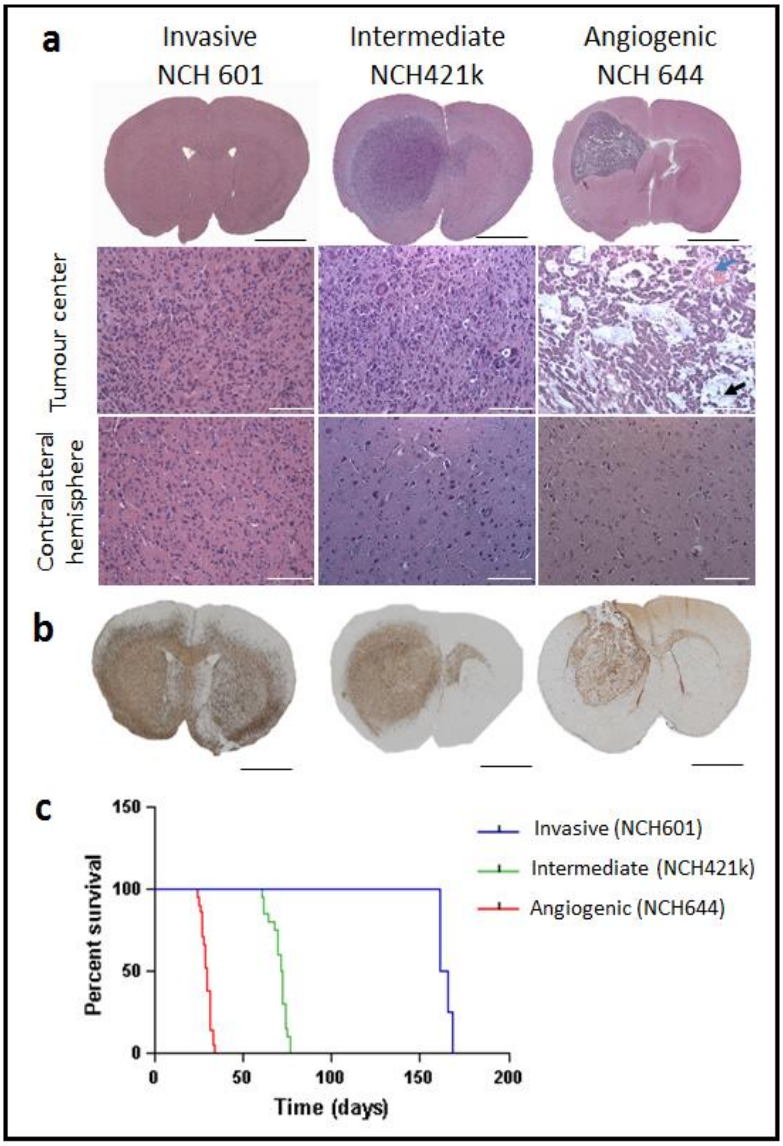

Suppl. Fig. 2. Phenotypes of glioma stem-like cell-derived xenografts (Related to **Fig. 1&2**). Glioma stem-like cultures were implanted intracranially in NOD/SCID mice. The developed tumours were classified into three phenotypes: invasive, intermediate and angiogenic. **A** Hematoxilin/Eosin staining showing tumour center and contralateral hemisphere of xenografts. Examples are shown for NCH601, NCH421k and NCH644. Blue arrow points to vessel with microvascular proliferation, black arrow shows necrosis. White and black scale bars represent respectively 100µm and 1mm. **B** Human-specific nestin (NCH601, NCH421k) or vimentin (NCH644) staining was used to detect tumour cells. Scale bars represent 1mm **c** Kaplan-Meier survival curve of stem-like cell line-derived xenografts (NCH421k and NCH644 n=20; NCH601 n=4).

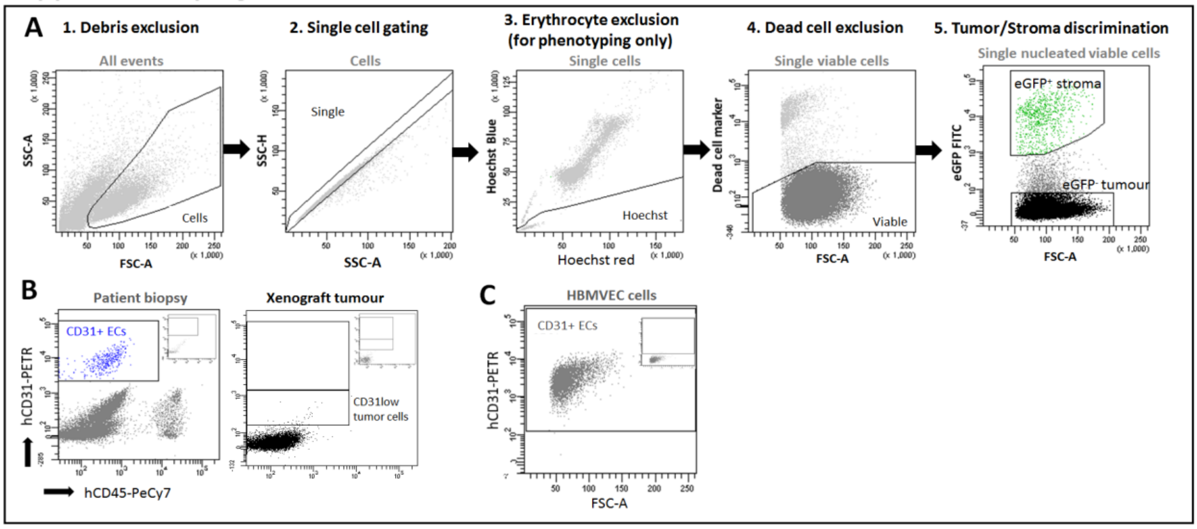

Suppl. Fig. 3. Gating strategy for sorting and multicolor phenotypic analysis (Related to **Fig. 3-6**). **A** Step-by-step gating strategy for FACS analysis is shown for the intracranial P3 xenograft in eGFP$^+$ NOD/SCID mice. The same strategy was used for all subsequent experiments using single viable cells, with exclusion of cell debris and erythrocytes. (**1)** Cells were distinguished from debris on the flow cytometric profile based on the Forward Scatter (FSC) and Side Scatter (SSC). (**2)** Cell doublets and aggregates were gated out based on their properties displayed on the SSC area (SSC-A) versus height (SSC-H) dot plot. (**3)** Dead cells were recognized by their strong positivity for the dead cell discrimination marker. (**4)** For multicolor phenotyping, erythrocytes were excluded by applying a 'Hoechst' gate on the 'Hoechst Red'/'Hoechst Blue' dot plot in the linear scale. Hoechst staining was omitted for cell sorting due to increased toxicity (**5)** In xenografts, human tumour cells were recognized as the eGFP negative population compared to the eGFP positive mouse host cells. **B** Comparison of CD31 expression in patient biopsy and tumour cells of a glioblastoma xenograft (T341). CD31+ **C**. CD31 expression in human brain microvascular endothelial cells (HBMVECs). Although CD31 staining was occasionally detected in a limited number of glioma cells (B; CD31 low tumor cells), the expression level was 10-100 times lower compared to CD31positive stromal cells in patient biopsies (B; CD31+ ECs 'blue') and HBMVECs (C).

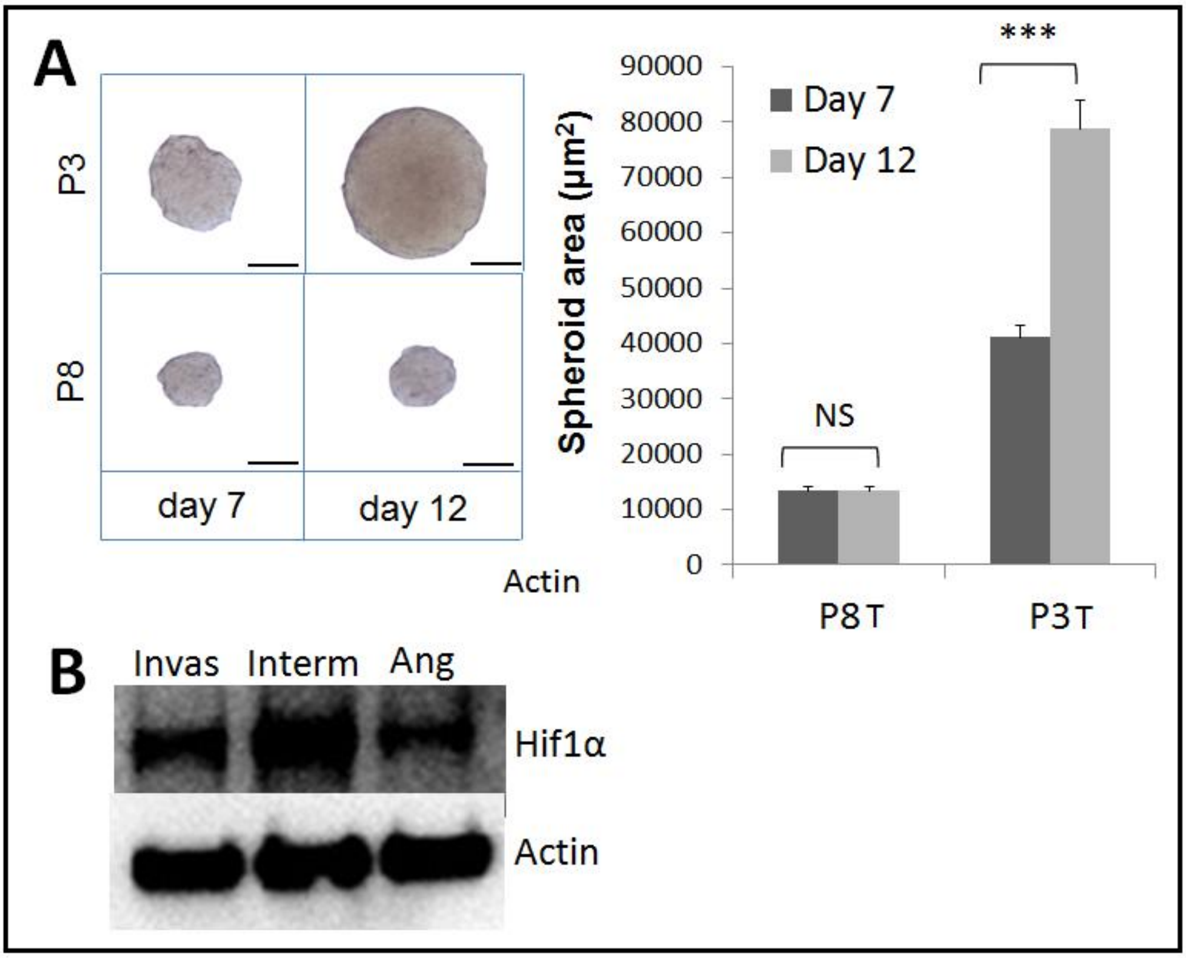

Suppl. Fig. 4. In vitro spheroid cultures. A. (Related to **Fig. 4)** Stroma-free spheroids were assessed for their growth capacities in vitro during 14 days in culture. Size measurements were performed at day 7 and 14. Invasive P8 spheroids hardly proliferated in vitro, whereas P3 intermediate spheroids displayed significant growth over the 14-day-culture. Scale bars represent 100µm; ***pvalue<0.001, n=20 per tumour. **B**. **(**Related to **Fig. 5)** Western Blot analysis showing the HIF1α protein present in organotypic spheroids in vitro of the angiogenic (Ang), intermediate (Interm) and Invasive (Invas) tumours.

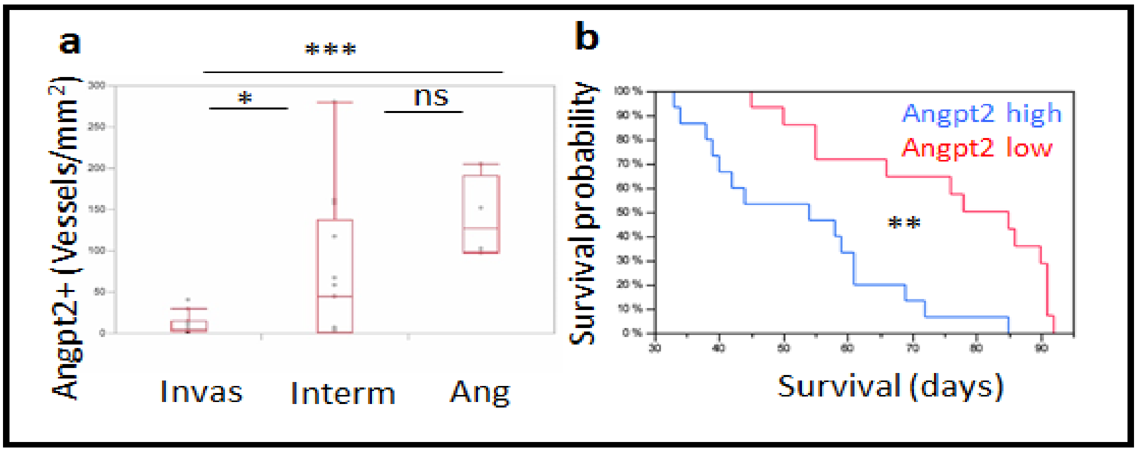

Suppl. Fig. 5 Analysis of Angiopoietin 2 expressing blood vessels (Related to **Fig. 6D). A** Quantification of Angiopoietin 2 (Angpt2) positive vessels confirmed significant differences between intermediate and angiogenic tumours versus invasive tumours. Analysis was performed for invasive (P8, T101, T185, T233, T239, T251), intermediate phenotypes (P3, T16, T238, T341, T434, NCH421k) and angiogenic (P13, NCH644) tumours. **B** Kaplan-Meier survival curve of xenotransplanted mice based on the Angpt2 expression (low/high) in blood vessels; p values were calculated with the Wilcoxon signed-rank test; *pvalue<0.05, **pvalue<0.01, ***pvalue<0.001.

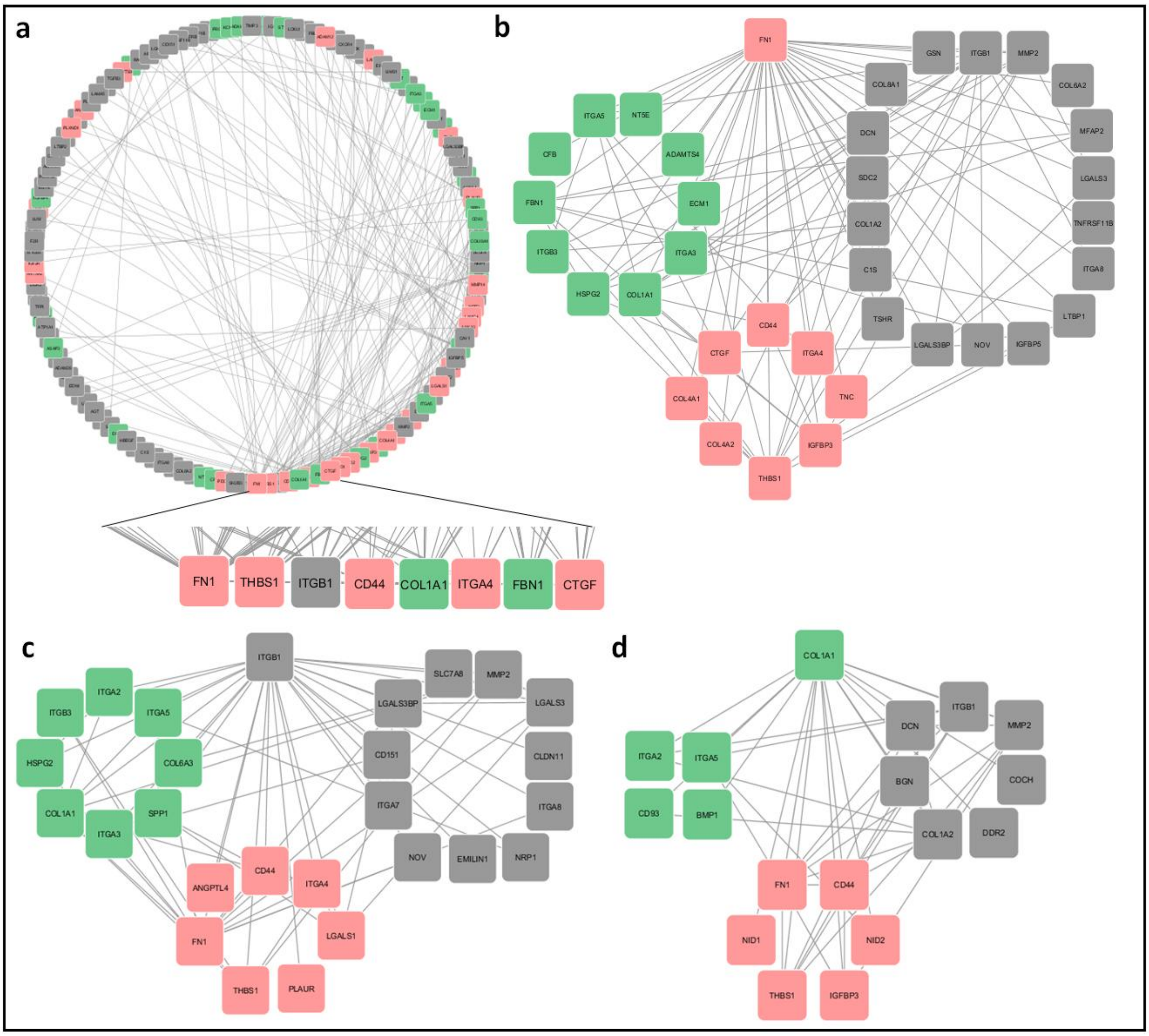

Suppl. Fig. 6. Selected networks for putative protein-to-protein interactions between tumor cells and ECs. A Direct protein-to-protein interactions between tumour and ECs were extracted from the network and displayed on circular layout sorted by degree of interactions (edges). Genes having highest number of interactions are highlighted. **B-D** Selected networks of protein-protein interactions for FN1 (b), ITGB1 (c) and COL1A1 (d) and their direct partners. Selected genes are displayed with their first neighbours grouped by category. Only the direct interactions between molecules upregulated in tumor and ECs are shown.

Suppl. Table 1. Clinical patient data and copy number aberrations of corresponding human glioblastoma biopsies.

Genomic aberrations as identified by aCGH are shown for all patient glioblastomas and cell lines usd in the study [++ = amplification (Log2 Ratio>2), + = gain (Log2 Ratio >0.35), - = loss (Log2 Ratio < -0.35), -- = deletion (Log2 Ratio < -1)]. Verhaak glioma subtypes classification (2) for patient biopsies based on the score for Clanc distance is presented when available.

Patient biopsy	Age	Sex	Chromosomal aberrations –Biopsy	Tumor subtypes
T16	52	F	++[EGFR , MDM2], +7q, - [Chr6q, Chr10, Chr11, 13q12-q32.2], --CDKN2A/B	Neural
T101	60	M	++[EGFR, MDM2] +Chr7, -[3q, Chr4, Chr10, Chr11, Chr15], --CDKN2A/B	Mesenchymal
T185	76	F	++EGFR, +Chr7, -10q, --CDKN2A/B	Mesenchymal
T233	43	F	++[EGFR, 2q34] +[1q21.2-24.2, Chr7, Chr19, Chr20], -[9p21.3 -21.1, Chr10], --CDKN2A/B	na
T238	41	M	+Chr7, -[6q, 9p-p21.1,Chr10, Chr13]	na
T239	79	M	+[Chr7, Chr19], -[1p-p35.3, 9p24.2-p23, 9p21.3-p21.1], --CDKN2A/B	na
T251	43	F	++[EGFR, 2q34], +[1q21.2-24.2, Chr7, Chr19, Chr20], -[9p21.3 -21.1, Chr10], --CDKN2A/B	na
T341	75	F	++[PDGFRA, 7p21.1, EGFR, 7q21.1-22.2, 17p12], 1[Chr7, Chr16, Chr19], -Chr10, --CDKN2A/B	na
T434	54	M	++[EGFR, NF1, MDM2], +[7p,20], -[4p, 6q26-qter, 9p, 9q22.32-qter, 10, 14q13-qter]	na
P3	64	M	+ [Chr 7, Chr19, 20q], -[1q42-q43, Chr9, Chr10, 20p] –[PIK3R , CDKN2A/B]	Mesenchymal
P8	64	F	++EGFR, +[Chr7, 8q24], -[6q22-q24, Chr10, 13q13.3-q33.3, 18q21.2-q22.4], --CDKN2A/B	Neural
P13	UN	F	+(Chr7, Chr19, Chr20),<br>-(6q16.2-16.3, Chr10, 17q12), --CDKN2A/B	Neural
Cell Line				
NCH421k	66	M	++[PDGFRA, MYC, CDK4], +[1p31.1-q43, 5q1-q22.2, 16p], -[2q, 3p-q13.31, 8p, 9p, Chr10, 13q]	Proneural
NCH644	67	F	++MYC, +[5q34-qter, EGFR, 8p, 8q-8q22.3, 20q11.23-qter, Chr21, Chr22], -[5q32.2-q34, 12q24.12-q24.32, 13q-q31.1, 18q, Chr19]	Proneural
NCH465	63	M	--[CDKN2C, CDKN2A/B], -10 (complex genome)	na
NCH601	76	M	++MycN, +7,--CDKN2A/B, -[4, 5, 6, 8, 10, 18]	na
NCH660h	74	F	+[5, 7, 12, 14, 16, 17, 21], --[CDKN2A/B], –[2, 6]	na

UN= unknown

Suppl. Table 2. Glioblastoma xenografts generated in NOD/SCID mice.

All xenografts used in the study are shown. For the development time, the mean time to sacrifice (days +/- SEM) and the number of mice per group (*n*) are indicated.

**Xenograft**	**Model**	**Phenotype**	**Generations after serial transplantation**	**Number of animals**	**Development time (NOD/Scid mice)**		
					Generation	Survival Days	Number of Mice
T16	Spheroid	Intermediate	6	66	G1	138+/-2	4
					G2	100.8+/-11.1	17
					G3	71.2+/-0.4	5
					G4	76.1+/-7.1	8
					G5	72.8+/-3.6	13
					G6	75.8+/-8.4	19
T101	Spheroid	Invasive	7	44	G1	111.8+/-4.3	10
					G2	117.3+/-3.1	7
					G3	104.9+/-15.1	8
					G4	104.2+/-1.5	4
					G5	101+/-1.5	6
					G6	93	5
					G7	107.7+/-1.5	4
T185	Spheroid	Invasive	3	17	G1	143.3+/-1.2	6
					G2	107+/-2	4
					G3	83+/-5.3	7
T233	Spheroid	Invasive	2	6	G1	152+/-7	3
					G2	107.7+/-4	3
T238	Spheroid	Intermediate	3	10	G1	140+/-2	3
					G2	155+/-8.5	2
					G3	84.4+/-4.9	5
T239	Spheroid	Invasive	1	3	G1	139.3+/-4.6	3
T251	Spheroid	Invasive	3	19	G1	122.5+/-1.7	4
					G2	86+/-4.2	8
					G3	71+/-1.5	6
T341	Spheroid	Intermediate	2	9	G1	58.5+/-0.6	4
					G2	54.6+/-6	5
T434	Spheroid	Intermediate	2	8	G1	61	3
					G2	45	5
P3	Spheroid	Intermediate	6	38	G1	42	3
					G2	48.4+/-1.7	8

					G3	41.3+/-0.5	6
					G4	43.6+/-0.5	5
					G5	39.4+/-0.7	11
					G6	40+/-0.7	5
P8	Spheroid	Invasive	3	31	G1	65.5+/-1	4
					G2	61+/-5.2	18
					G3	52.2+/-0.8	9
P13	Spheroid	Angiogenic	3	31	G1	56	1
					G2	41.9+/-2.4	20
					G3	40.3+/-2.7	10
NCH421k	Neurosph.	Intermediate	1	20	G1	70.4+/-4.8	20
NCH644	Neurosph.	Angiogenic	1	21	G1	29.3+/-2.6	20
NCH465	Neurosph.	Invasive	1	6	G1	133.7+/-12.6	6
NCH601	Neurosph.	Invasive	1	4	G1	165+/-1.7	4
NCH660h	Neurosph.	Invasive	1	8	G1	258.5+/-16.6	8

Suppl. Table 3. List of antibodies used in the study.

Epitope	Conjugate	Species reactivity	Clone	Supplier	Concentration used/test*
CD31	Dy590 (PE-TR)	human	MEM-05	Immunotools	F: 10µl/test
CD31	APC	mouse	390	eBioscience	F: 2.5µl/test
HIF1α	-	human	54/HIF-1α	BD transduction lab 610959	WB:1/1000 = 250 ng/ml
HIF2α	-	human	Ep190b	Novus biological NB100-132	WB:1/2000 =500ng/ml
Actin	-	human/mouse/rat	C4	Milllipore MAB 1501	WB:1/6000
CD31	-	mouse	390	Millipore	IHC:1/200
CD31	Dy590 (PE-TR)	human	MEM-05	Immunotools	10µl/test
CD45	PE-Cy7	human	HI30	Immunotools	5µl/test
MCT4	-	mouse/rat/human	H-90	Santa Cruz Biotechnology	IHC:1/200
Nestin	-	human	10C2	Millipore	IHC:1/200
Vimentin	-	mouse/rat/human	EPR3776	Epitomics	IHC:1/200
Angiopoietin 2	-	mouse/rat/human	PA5-27297	ThermoScientific	IHC: 1/200
Ki67	-	mouse/rat/human	SP6	ThermoScientific	IHC: 1/100
Thrombospondin 1	-	mouse/rat/human	A6.1	Thermo scientific, MA5-13398	IHC: 2ug/ml
Anti-mouse -IgG	HRP	mouse		GE Healthcare LNA931V/AG	WB: 1/10 000
Goat anti-rat IgG	Alexa Fluor 555	rat		Invitrogen	IHC:1/1000
Anti-mouse IgG	HRP	mouse		Dakocytomation	Kit concentration
Anti-rabbit IgG	HRP	rabbit		Dakocytomation	Kit concentration
Anti-Mouse IgG	Biotinylated	horse		Vestor labs, BA-2000	IHC:0.2ug/ml

F = Flow cytometry (test 10^6 cells/100µl); WB = Western Blot; IHC = Immunohistochemistry

Suppl. Table 4. Comparison of differentially expressed genes (DEGs) between angiogenic P13, intermediate P3 and invasive P8 tumour cells.

Differentially expressed genes between sorted tumour cells of the angiogenic (P13), intermediate (P3) and invasive (P8) tumour cells were determined with the eBayes (Limma) linear model. Cut-offs were set up for FDR<0.01 and abs(FC)>=2. Common and unique genes were established by the SUMO software Venn diagram analysis (http://angiogenesis.dkfz.de/oncoexpress/software/) (not shown). Gradually increased and decreased genes were extracted as genes common in three DEG comparisons (P13vP8, P13vP3 and P3vP8) for upregulated and downregulated genes respectively. Genes upregulated in angiogenic and intermediate versus invasive tumors were extracted from common upregulated genes between P13vP8 and P3vP8. Genes specific for angiogenic tumors were extracted from common upregulated genes between P13vP8 and P13vP3 comparisons. Genes upregulated in invasive and intermediate tumors versus angiogenic tumors were extracted from common downregulated genes between P13vP3 and P13vP8 comparisons. Genes specific for invasive tumors were extracted from common downregulated genes between P13vP8 and P3vP8. Unique genes specific for each comparison are not shown.

Gradual increase Ang>Int>Inv	Up in Ang and Interm v. Inv	Up in Ang only	Gradual decrease Ang<Int<Inv	Up in Interm and Inv	Up in Invas only
P8 P3 P13	P8 P3 P13	P8 P3 P13	P8 P3 P13	P8 P3 P13	P8 P3 P13
**91**	**425**	**508**	**51**	**510**	**255**
ADAMTS18	A2M	ABCA8	AFF3	ABAT	ABCG1
AGPHD1	ABCA5	ABCC3	AGTPBP1	ABCD3	ACTG1P4
AGT	ABCC4	ADAM12	ALCAM	ABHD3	ADCK3
ANK3	ABCD1	ADAM19	ANKRD20A11P	ACADM	ADCY1
ANXA11	ABCD2	ADAM28	ATCAY	ACBD7	ALDH1L2
BCL11A	ACSS2	ADAM32	C5orf54	ACVR2A	ALPL
C10orf107	ACSS3	ADAMTS9	CACNA1D	ADAM22	AMPH
CAV1	ADA	ADARB2	CD24	ADAM23	AMY1A
CAV2	ADAMTS3	ADM	CLVS2	ADAMTS5	AMY2B
CHSY3	AHNAK	ADORA2A	COL20A1	ADCYAP1R1	ANK1
CNN2	AK4	ADORA2B	CPM	AFAP1L2	ANKRD20A3
CNR1	AKAP7	AKT1S1	CYTL1	AGMO	ANKRD20A5P
COPZ2	ALDH7A1	ALKBH7	DIRAS2	AKD1	ANKRD20A8P
CXCR4	ALDOC	ALPK1	DLL3	AKT3	ANXA2R
CYB5R2	AMOT	ANGPT2	DRP2	ALDH1L1	ARSE
EGLN3	ANGPTL1	ANKS1B	EDA2R	AMACR	ASB5
EYA4	ANTXR2	ANO1	EGFR	ANKH	ATP10B
FAM129A	ANXA1	ANXA2	FBN3	ANKRD12	ATRNL1
FAM198B	ARHGAP18	ANXA2P2	FGF14	ANKRD20A12P	BCAS1
FAM84B	ARHGAP20	AP2M1	GIMAP2	ANKRD36	BEST1
FGF12	ARID1B	APLN	HOXA2	ANKRD36B	BRD9
FILIP1L	ARL4C	APOC1	KCND2	ANKRD36C	BRSK2
FLNC	ARSA	AQP1	KIAA1244	ANKRD45	BTN1A1
FRMD4B	ATP10D	AQP5	KLRC4-KLRK1	ANKRD6	C11orf41
FSTL1	ATP11C	ARHGAP26	LOC150622	AP3B2	C2orf80
GALNT4	AXL	ARHGEF3	LOC283174	APBB2	C5
GJA1	B3GAT2	ARPC1B	LRRC4	APCDD1	C6orf123

GLRX	BDH2	ARRDC3	LRRFIP1	APOD	C8orf46
GPD1L	C10orf11	ARSJ	LYPD1	APOL4	CABLES1
GPX8	C11orf63	ATP5SL	MTBP	APOL6	CACNA1A
GXYLT2	C11orf70	AVPI1	MTSS1	ARAP2	CACNA2D2
HK2	C14orf101	B3GNT2	NALCN	ARHGEF6	CACNB3
ID1	C14orf169	B3GNT5	NCAM2	ARMCX4	CADM3
ID3	C14orf23	B4GALT1	NKAIN4	ART3	CBWD1
INPP4B	C14orf38	BASP1	NOL4	ASCC3	CBWD3
KCNJ6	C1orf201	BCAT2	RAPGEF4	ASCL1	CBWD5
KCTD12	C1orf85	BCL2L12	RGS7BP	ASPA	CCDC136
KDELR3	C1QTNF6	BDNF	RN5S156	ASRGL1	CCND1
KIAA1199	C1R	BGN	RPS6KA5	ATAD5	CD59
KIAA1598	C1RL	BHLHE41	SCG3	ATP13A4	CDK14
LGALS3	C1S	BICC1	SCN2A	ATP13A5	CDKL4
LIN7A	C3AR1	BID	SERPINE2	ATP1A2	CELF5
LOX	C6orf72	BMP2	SNTB1	ATP1A3	CENPV
LYN	C8orf4	BMP6	SNX22	ATP2B4	CER1
MGC45800	C9orf64	BMPER	SULF2	B3GALNT1	CHML
MOXD1	C9orf72	BNIP3	TNR	B3GALT2	CHRNA4
MYOF	CA12	BOC	TOX3	B3GALT5	CKMT1A
NAALADL2	CA13	BTBD11	WWC1	B4GALT6	CLK4
NPAS2	CALCRL	C10orf10	ZDHHC22	BAI3	CNPY1
NPNT	CAPRIN2	C10orf122	ZNF415	BBS2	CNTLN
ODZ2	CASP6	C10orf125	ZSCAN16	BCAN	CNTNAP5
OTX1	CASP8	C14orf57		BMPR1B	CNTRL
PCDH20	CASQ1	C1QL2		BRCA1	CPEB3
PCOLCE	CAST	C1QTNF1		BST2	CYP3A5
PDE8B	CCDC109B	C1QTNF9B-AS1		BTG2	DARC
PDGFC	CCNA1	C2CD4A		BVES	DDX11L2
PHACTR2	CD109	C2orf40		C12orf76	DDX25
PLEKHA5	CD276	C3orf58		C17orf75	DEM1
PLIN2	CD2AP	C3orf80		C21orf62	DOCK3
PLXDC2	CD97	C8orf22		C4A	DUSP26
PQLC3	CD99	C8orf48		C4orf21	DYNC1I1
PROM1	CDC42EP1	CA9		C5orf30	EFNA3
PTCH2	CDH11	CALB1		C5orf63	ELAVL4
PTGR1	CDON	CALB2		C5orf64	ELFN2
PTRF	CDR1	CALHM2		C6orf130	EPB41
RAPGEF5	CEBPB	CAV3		CA8	EPHB1
RBMS3	CEBPD	CCDC102B		CACNB4	ERAP2
RTTN	CELSR1	CCDC86		CCDC144A	ETS2
SCD	CEP85L	CCL2		CCDC15	EXOSC3
SDC2	CFI	CD226		CCDC18	FABP6
SEMA3A	CHI3L1	CD248		CD200	FAM135B
SGK1	CHMP2B	CD34		CD82	FAM149A
SLC10A4	CHRNA5	CD44		CDC42EP3	FAM218A
SLC27A3	CHST7	CD55		CDC7	FAM27E3
SNAI2	CLDN10	CDH5		CDH1	FAM89A
SRPX2	CLMP	CHCHD1		CDKL5	FBLL1
ST6GALNAC5	CLN5	CHCHD10		CEP152	FLJ13197
ST8SIA3	CLYBL	CHRDL1		CEP44	FLJ35776
STC1	CNGA3	CHRM3		CHD7	FLJ38109
STMN2	COBLL1	CHRNA9		CHDH	GABBR2
TGFB1	COCH	CITED2		CHGB	GADD45G
TLE1	COL1A2	CLDN11		CHRNB2	GAL3ST1
TLR4	COL4A1	CLIC6		CLIP2	GATSL2
TMBIM1	COL4A2	CLSTN2		CMTM5	GBAP1
TMTC1	COL5A2	CMKLR1		CNOT6L	GBX2
TNFAIP3	CPEB2	CMTM7		CRB1	GFRA1
TNFSF4	CPNE8	CNIH3		CREBZF	GLDN
TOX	CPQ	CNTNAP3		CRIPAK	GLYATL2
TRPM3	CRABP1	COLEC12		CRISPLD1	GNG3
VEGFA	CRYBG3	CPNE4		CRMP1	GPR12
ZNF385D	CSDA	CPPED1		CRYZ	GPR139
	CTNNA3	CPZ		CSPG4	GPR17
	CTSZ	CRABP2		CSPG4P5	H2AFY2
	CXCL14	CRH		CTH	HAPLN1
	CYB5A	CRISPLD2		CTPS2	HECW2
	CYB5R1	CRYM		CTSK	HIP1
	CYFIP1	CSRP1		CXorf1	HLA-DMA
	DDR2	CTGF		CXXC4	HMP19

	DENND1B	CUBN		CYFIP2	HOXB8
	DHCR7	CUZD1		DBX2	HOXB9
	DIAPH2	CYSTM1		DDX60	HOXC9
	DLC1	DAB1		DDX60L	HOXD10
	DLG2	DBC1		DENND2A	HRASLS
	DNAH11	DCBLD2		DKFZP434I0714	HS3ST1
	DNAJC3	DCN		DLL1	IFITM1
	DNAJC4	DDIT4		DNAJB4	IFITM10
	DNALI1	DDX21		DNER	IGSF21
	DPP4	DHRS3		DNM3	IL33
	DTWD2	DIO2		DOCK11	JHDM1D
	DYNLT1	DKK2		DPP6	JPH3
	E2F5	DLX1		DTX4	JPX
	ECHDC2	DLX2		EFTUD1	KANK1
	EFHA2	DNAH14		ELF2	KCNA6
	EFNB2	DNAJA4		EML6	KCNB1
	EFR3B	DNAJC12		ENHO	KIAA1755
	EGR1	DNAJC5B		ENTPD1	KIAA2022
	EGR2	DNM3OS		ERBB3	KLHL9
	EHD2	DNMBP		ETNK2	KLRC1
	EIF4E3	DOCK5		ETS1	KLRC4
	EIF5A2	DOK5		EVI5	LINC00537
	ELF4	DOK6		FAM102B	LNX1
	ELL2	DPCD		FAM106A	LOC100128288
	EMC2	DPP10		FAM111A	LOC100130876
	EMILIN1	DRD2		FAM111B	LOC100132356
	EMP2	DUSP23		FAM126A	LOC100133106
	EMP3	EBF2		FAM131B	LOC100506965
	ENC1	ECHS1		FAM161A	LOC389834
	EPAS1	EDN1		FAM167A	LOC400590
	EPDR1	EDN3		FAM169A	LOC441179
	EPG5	EFHC2		FAM40B	LOC646903
	EPHA3	EFNA4		FAM70A	LOC648987
	EPHA5	EFNA5		FANCI	MARCH1
	EPHB4	ELAVL2		FANCM	MCF2L2
	ERBB4	ELK3		FAXC	MIR600HG
	ERO1L	EMX2		FBXO21	MIR95
	F2R	ENO4		FBXO48	MPPED2
	F2RL2	ENPP2		FBXO9	NETO1
	FAH	ENTPD3		FCHO2	NFE2L3
	FAM109B	EPHA2		FGD6	NIPAL2
	FAM59A	ERCC2		FHOD3	NKAIN1
	FAM82A2	ERRFI1		FLJ36840	NNAT
	FAM86B1	EXTL3		FLJ44124	NPTX2
	FAM86DP	FAM101B		FMN2	NRG1
	FAP	FAM115C		FMOD	NRSN1
	FAR2	FAM129B		FPGT	NS3BP
	FARP1	FAM180A		FREM1	NT5E
	FAT1	FAM24B-CUZD1		FRY	NTRK2
	FBLN5	FAM84A		GAB2	NUDT7
	FBLN7	FBXW7		GABBR1	NXN
	FBN2	FEZF2		GAL3ST4	OLFM1
	FBXL7	FGL2		GALR1	OPCML
	FBXO17	FHIT		GATS	OR4N2
	FECH	FLJ44635		GBP1	PAIP2B
	FGFRL1	FLNB		GBP3	PALM2-AKAP2
	FHL3	FLRT3		GDAP1L1	PAX3
	FN1	FNDC3B		GDPD1	PCDHB17
	FOXG1	FOSL2		GDPD2	PCDHB3
	FREM2	FRAS1		GJC1	PCSK2
	FRRS1	FZD8		GLMN	PIK3R1
	FRZB	GABRA3		GNAO1	PIP5K1B
	FUCA2	GABRA4		GOSR1	PLA2G5
	FUT9	GABRA5		GPR156	PLAC1
	FZD7	GABRB1		GPR37L1	PLCL1
	GALC	GABRB3		GPR39	PLD5
	GALM	GABRG3		GPR75	PLEKHA7
	GCA	GAD1		GPRASP1	PRKCZ
	GDF15	GATA6		GRAMD3	PRKX
	GFPT2	GCNT1		GUCY1B3	PTPRD
	GLIPR1	GLIS3		GUSBP1	PTPRO

	GM2A	GLRA2		GUSBP11	RAB24
	GNPNAT1	GNA14		GYG2	RAB3C
	GPC6	GNAS		HACE1	RAB3IP
	GPR98	GPD1		HCG8	RARB
	GPX7	GPR124		HERC5	RASSF10
	GRB10	GPR158		HERC6	RGPD2
	GRB14	GRAMD1B		HES5	RN5S403
	GULP1	GREB1		HES6	RND1
	GYPC	GRIK1		HEY2	RPL39L
	H1F0	GRIN2A		HFM1	RPS14
	HIST1H1A	GRM3		HIBCH	RPSAP58
	HIST1H2AJ	GRM7		HIST1H2BB	RRAGD
	HIST1H2BM	GRPR		HIST1H2BC	RTN4RL2
	HIST1H4F	GSN		HNMT	SCARNA17
	HRH1	GSTO1		HOXA4	SCN4B
	HS3ST3A1	GSTT1		HOXA9	SCXA
	HS3ST3B1	HBEGF		HOXB2	SERTAD4
	HSD17B6	HK1		HOXD3	SIM2
	HSD17B8	HKDC1		HSD17B7P2	SIX1
	IFNGR1	HMCN1		ICA1L	SIX4
	IFT74	HPS1		IFI44	SIX6
	IL13RA1	HRH2		IGF1R	SLA
	IL13RA2	HSD17B14		IGSF5	SLC16A7
	IL17RA	HSPA12A		IL17RB	SLC44A5
	IL7	HSPB6		ITGA2	SLC8A3
	INTS6	HTRA1		ITGA6	SMPD3
	IQGAP1	ICAM3		KCNA2	SNHG1
	IQGAP2	IDE		KCNC4	SNORA54
	ITGA7	IFITM2		KHDRBS3	SNORD113-4
	ITPR3	IGF1		KIAA0226L	SNORD114-2
	KAL1	IGFBP3		KIAA1147	SNORD114-26
	KCNMB4	IGFBP5		KIAA1161	SNORD30
	KHNYN	IGFBP6		KIAA1324L	SOD3
	KIF13B	IMMP2L		KIF1A	SORCS3
	KITLG	INA		KIF5A	SOX1
	KLHL13	IPO4		KLHL25	SOX11
	KLHL26	IRX3		KLRC3	STAG3
	KLHL4	ISOC2		KPNA4	STAMBPL1
	L3MBTL3	ITGA8		KSR1	STK32A
	LAMA2	ITGB1		LAMA4	STK32B
	LAMA5	ITIH5		LGR5	STMN4
	LAMP1	ITPRIP		LHFPL3	SUSD4
	LANCL3	JAG1		LIG3	TAS2R43
	LATS2	JUP		LIMCH1	TBX15
	LBH	KCNF1		LINC00461	TBX3
	LDB2	KCNIP4		LINC00526	TBX5
	LDLR	KCNJ2		LINC00597	TCL1A
	LGALS1	KDELR1		LINGO1	TEK
	LIMS1	KIAA1211		LNX2	TGFBR3
	LIPG	KIAA1456		LOC100129455	TMEM108
	LMAN1	KIF16B		LOC100130275	TMEM163
	LMF1	KIRREL		LOC100130428	TMEM98
	LMNA	KLF5		LOC100133315	TNS3
	LOC390940	KLHDC8A		LOC100505572	TPPP
	LPHN2	KLHL14		LOC349196	TRIM52
	LPPR4	KLHL32		LOC375295	TRIM67
	LRIG1	KRT75		LOC400940	TRMT12
	LRP10	LAMB1		LOC441204	TRNAF14P
	LRP1B	LAMC1		LOC442075	TTC9B
	LRRC16A	LAMP5		LOC728715	TTLL9
	LRRC17	LCTL		LPHN3	ULBP1
	LY75-CD302	LDOC1		LRP4	UPF3B
	MAB21L1	LEMD1		LRRC1	VAT1L
	MAGT1	LEPREL1		LRRC37A	VIPR2
	MALT1	LIMK1		LRRC37A2	VSTM2B
	MAP3K5	LOC100506718		LRRC37B	WASF3
	MAP7	LOC100506948		LRRC40	WDR67
	MAPK1IP1L	LOC151009		LRRC4C	WEE1
	MAPK4	LOC645261		LRRK2	WNT7B
	MDFIC	LOC654433		LRRN1	XYLT1
	MED23	LOXL1		LSM6	ZCWPW1

	METTL7B	LOXL2		MAMLD1	ZDHHC11
	MGLL	LOXL4		MAP3K3	ZIC1
	MGP	LPAR1		MAPT	ZIC4
	MID2	LPPR5		MASP1	ZNF154
	MINA	LRRC15		MAST1	ZNF233
	MITF	LRRC3B		MATN2	ZNF257
	MKNK1	LRRTM3		MB21D2	ZNF300
	MMP14	LSM10		MBNL3	ZNF454
	MOB3B	LTBP1		MEGF10	ZNF461
	MORC4	LUM		MEOX2	ZNF528
	MPV17L	LY96		MFAP3L	ZNF578
	MRAP2	LYPD6B		MFNG	ZNF610
	MRC2	MAEL		MFSD6	ZNF665
	MTTP	MAFB		MGAT5	ZNF678
	MYH9	MAMDC2		MIR186	ZNF679
	MYO1E	MAN2A1		MIR2964A	
	MYO5B	MB21D1		MIR9-1	
	NAAA	MCHR1		MMD2	
	NAPRT1	MCTP2		MTF2	
	NDUFS5	MCU		MYBL1	
	NEDD9	MDGA2		MYO5A	
	NEK9	MDK		MYO5C	
	NEUROD1	ME1		MYT1	
	NFATC1	MED12L		NAIP	
	NHSL1	MFSD2A		NAP1L3	
	NIPSNAP3A	MGC39372		NCKAP5	
	NMNAT3	MKNK2		NCOA7	
	NMRAL1	MMP2		NDRG2	
	NMU	MRGPRF		NEB	
	NOS2	MRI1		NECAB1	
	NOTCH3	MRPL33		NEK1	
	NPC2	MRPL34		NF1	
	NRCAM	MRPS12		NF1P3	
	NRP2	MRPS15		NKD1	
	ODZ1	MSRB3		NKX2-2	
	ODZ3	MSTN		NMD3	
	ODZ4	MT1A		NMI	
	OGFRL1	MT1E		NPPA	
	OSBPL10	MT1F		NRARP	
	OSBPL3	MT1G		NRXN1	
	OSMR	MT1X		NRXN2	
	OTX2	MYADM		NTRK3	
	OXR1	MYL9		OCIAD2	
	PALLD	MYO16		OLIG1	
	PALMD	MYO1B		OLIG2	
	PAM	NABP1		OMG	
	PARK2	NCEH1		OPHN1	
	PCDH17	NDRG1		OVOS	
	PCDH19	NDUFA4L2		OXCT1	
	PCDH9	NEDD4L		PAK3	
	PDE10A	NGFR		PARP12	
	PDE3A	NHSL2		PARP14	
	PDGFRA	NID1		PCDHB1	
	PDLIM1	NID2		PCDHB12	
	PDLIM4	NNMT		PCDHB13	
	PDPN	NOC3L		PCDHB14	
	PFKFB3	NOV		PCDHB15	
	PGM5	NPR1		PCDHB16	
	PHACTR1	NPY		PCDHB18	
	PHF11	NR1D1		PCDHB2	
	PIGB	NR1H2		PCMTD1	
	PIGN	NR2E1		PDE1C	
	PIP4K2A	NRP1		PDSS2	
	PITX2	NRXN3		PEAK1	
	PLA2G16	NTAN1		PHF16	
	PLA2G7	NXPE2		PHYHIPL	
	PLAUR	NXPH4		PID1	
	PLCH1	OAS1		PIGK	
	PLEKHG1	OAS3		PIK3C2B	
	PLIN3	OGDHL		PKIA	
	PLOD2	OLFML3		PKN2	

	PLP2	ONECUT2		PLEKHG3	
	PLSCR4	OXTR		PLEKHH1	
	PLXND1	P4HA1		PLEKHH2	
	PMAIP1	P4HA2		PLK1S1	
	POU3F4	PABPC5		PLLP	
	PPIC	PAWR		PLP1	
	PPP1R1C	PCDHB7		PLXNC1	
	PPP2R3A	PCOLCE2		PNISR	
	PRDM5	PDE7B		POLE4	
	PREX2	PDGFD		PPFIBP2	
	PRKCQ	PDGFRB		PPM1K	
	PRPS2	PDLIM7		PPM1L	
	PRR5L	PER1		PPP1R14C	
	PRRG1	PERP		PPP1R9A	
	PTCHD1	PFN1P2		PPP2R2B	
	PTPN14	PGAM1		PRKAR2B	
	PTPRE	PI4K2A		PSD2	
	PTPRK	PKIG		PTBP2	
	PVRL3	PLA2R1		PTCH1	
	PXMP4	PLAGL1		PTGIS	
	RAB11FIP2	PLBD1		PTGS1	
	RAB32	PLCB4		PTP4A3	
	RAB3D	PLD1		RAB11FIP4	
	RANBP3L	PLEKHG5		RAB33A	
	RASA3	PLOD1		RAB39B	
	RASGRP1	PLXNA1		RABGGTB	
	RBPMS	PMEPA1		RAP1GAP	
	RDH10	PODXL		RBMX	
	REEP3	POPDC3		RFX4	
	RETSAT	POSTN		RGNEF	
	RFFL	PPA1		RICTOR	
	RGS6	PPAP2C		RIMKLB	
	RNF149	PPDPF		RIT2	
	RNF217	PPP1R15A		RLBP1	
	ROR1	PPP1R3B		RN5S104	
	RORB	PPP4R1L		RN5S196	
	RYR3	PRDM1		RN5S295	
	SCG2	PRDX3		RN5S97	
	SDC4	PRKCDBP		RNF144A	
	SEMA6A	PRKCH		RNF182	
	SERPINB1	PROX1		RNU7-35P	
	SERPINF1	PRR7		RPAP2	
	SFRP4	PRRX1		RTN4IP1	
	SGK3	PRRX2		RUNDC3A	
	SGMS2	PRSS35		S100A11	
	SLC12A2	PSTPIP2		S100B	
	SLC20A2	PTCHD4		SAMD9	
	SLC22A5	PTEN		SAMD9L	
	SLC25A37	PTGES		SATB1	
	SLC26A7	PTHLH		SCARNA5	
	SLC2A10	PTPRM		SCARNA6	
	SLC39A8	PTPRT		SCARNA7	
	SLC40A1	PTX3		SCARNA9L	
	SLC4A10	PVRL2		SCG5	
	SLC7A11	PXDNL		SCN1A	
	SLC7A2	PYROXD2		SCN3A	
	SLC9A9	RAB3A		SCN8A	
	SLCO1C1	RAB7A		SDC3	
	SLITRK3	RALGAPA2		SEMA5A	
	SLMO2	RAMP2		SEMA5B	
	SMAD9	RAP2B		SEPT3	
	SMG8	RASSF3		SERF1A	
	SNAP23	RASSF8		SEZ6	
	SOCS6	RBPMS2		SEZ6L	
	SOD2	RCAN2		SH3GLB1	
	SOGA3	RCN3		SHD	
	SORBS3	REC8		SHISA6	
	SP8	REEP4		SKAP2	
	SPOCK2	REEP6		SLC13A5	
	SRPX	RFPL4A		SLC15A2	
	STIM2	RFTN1		SLC16A4	

	STK3	RGS4		SLC19A3	
	STX7	RNASEK		SLC1A4	
	SUCLG2	RNF175		SLC24A3	
	TAAR3	RNLS		SLC2A13	
	TCIRG1	RPH3A		SLC37A1	
	TFCP2	RPL29		SLC38A3	
	TFPI	RPS24		SLC41A2	
	TGFB3	RPS6KA2		SLC46A3	
	TGIF2	RRAS		SLC4A8	
	TIAM2	RRP12		SLFN11	
	TIFA	RRS1		SLFN13	
	TIMP3	RXFP1		SLIT1	
	TMC7	SAMD5		SLITRK2	
	TMEM132B	SAT1		SMA4	
	TMEM144	SCARB1		SMA5	
	TMEM154	SCARF2		SMOC1	
	TMEM176A	SCFD2		SNORA22	
	TMEM47	SCGN		SNORD42A	
	TMTC2	SEL1L3		SNORD4B	
	TNFRSF11B	SELM		SNORD58A	
	TNFRSF1A	SEMA7A		SNORD94	
	TRIM59	SERPINE1		SOCS2	
	TRIM9	SERPINH1		SORBS2	
	TRIP6	SERPINI1		SOX13	
	TTC39C	SFRP1		SP100	
	TUBA1C	SFXN3		SPECC1	
	TWIST1	SGSH		SPSB4	
	ULBP3	SH2B3		ST3GAL5	
	ULK4	SHC1		ST3GAL6	
	USP25	SHKBP1		ST6GAL1	
	USP6NL	SHROOM2		STAG3L4	
	UTRN	SIK2		STK17B	
	VRK2	SIPA1L2		STOX2	
	VWA5A	SLC16A9		SULT1C4	
	WDFY2	SLC22A23		SYBU	
	WIPI1	SLC2A1		SYNE2	
	WWC2	SLC35G1		SYT17	
	WWTR1	SLC39A14		TADA2A	
	YAP1	SLC6A6		TAGLN3	
	ZDHHC2	SLC7A5		TAOK3	
	ZFAND4	SLC7A8		TAS2R19	
	ZNF132	SLCO2A1		TAS2R31	
	ZNF404	SLN		TDRKH	
		SMARCE1		TEFM	
		SNHG15		TEX9	
		SNORD115-32		TFAP2A	
		SNORD115-4		THEM4	
		SNORD115-40		THRB	
		SNORD115-44		TLCD1	
		SNORD115-5		TLE3	
		SNORD115-6		TLR3	
		SNORD49B		TM4SF1	
		SNRPB2		TMEM132C	
		SNRPN		TMEM169	
		SNTG1		TMEM229B	
		SORCS2		TMEM71	
		SPARCL1		TMLHE	
		SPOCD1		TMSB15A	
		SPON1		TNFAIP6	
		SPON2		TNK2	
		SREBF1		TOM1L1	
		SRM		TRAF3IP2	
		SRXN1		TRIM38	
		ST3GAL1		TSHZ3	
		ST3GAL4		TSPAN11	
		ST6GAL2		TSPAN12	
		ST6GALNAC4		TSPAN18	
		STAC		TSPAN7	
		STAC2		TXNIP	
		STOM		UNC13A	
		STXBP6		UNC80	

		SVIL		USP32P2	
		SYDE2		USP33	
		SYNM		USP45	
		SYNPO		UST	
		SYNPR		UTP6	
		TAGLN		VCAN	
		TBX1		VPS29	
		TBX2		VTRNA1-3	
		TCEA3		WSB1	
		TCF7L2		XAF1	
		TFAP2C		YPEL1	
		TGFBR2		ZC3H12B	
		THBS1		ZC3H6	
		THSD1		ZC4H2	
		THSD4		ZCCHC18	
		TIMM23		ZDBF2	
		TM4SF18		ZEB2	
		TMEM2		ZFP14	
		TMPRSS2		ZFP2	
		TNC		ZFP62	
		TNFSF10		ZFP82	
		TOX2		ZMAT1	
		TPD52L1		ZNF10	
		TPM2		ZNF167	
		TRABD2A		ZNF184	
		TRAF7		ZNF204P	
		TRAPPC3		ZNF214	
		TRGC2		ZNF221	
		TRNAP24P		ZNF229	
		TRNAS32P		ZNF256	
		TRNAU2		ZNF268	
		TSHR		ZNF277	
		TSHZ2		ZNF311	
		TSPAN14		ZNF326	
		TSPAN9		ZNF418	
		TTC23L		ZNF506	
		TTLL6		ZNF514	
		TUBA4A		ZNF521	
		TUBB2A		ZNF620	
		TUBB4A		ZNF626	
		UIMC1		ZNF642	
		UNC5B		ZNF643	
		UNC5D		ZNF644	
		USMG5		ZNF704	
		VIT		ZNF711	
		VTRNA1-2		ZNF730	
		WIF1		ZNF737	
		WNK4		ZNF781	
		WNT5A		ZNF846	
		WWP1		ZNF852	
		ZNF395		ZNF90	
		ZNF503		ZNHIT6	
		ZNF511		ZNRD1-AS1	
		ZNF518B		ZRANB2	
		ZNF593		ZSCAN23	
				ZSWIM5	
				ZZZ3	

Suppl. Table 5. DAVID analysis for differentially expressed genes (DEGs) between angiogenic (P13) versus invasive (P8) tumour cells.

Differentially expressed genes between sorted tumour cells of the angiogenic (P13) versus invasive (P8) phenotype were determined with the eBayes (Limma) linear model. Cut-offs were set up for FDR<0.01 and abs(FC)>=2. 2672 DEGs were divided into upregulated (FC>=2; 1393 genes) and downregulated genes (FC<=-2; 1279 genes). DEG lists were submitted to the DAVID® database (DAVID 6.7; http://david.abcc.ncifcrf.gov/) for functional enrichment analysis. Main significantly deregulated Gene Ontology (GO) terms, UniProt keywords and features are presented if the enrichment score for the annotation cluster was >2 with terms p value<0.05.

**Angiogenic (P13) vs. Invasive (P8) Upregulated**		**Angiogenic (P13) vs. Invasive (P8) Downregulated**	
Term	PValue	Term	PValue
**Enrichment Score: 15.7**		**Enrichment Score: 5.64**	
signal	2.32E-26	metal-binding	1.88E-08
signal peptide	4.32E-26	GO:0046872~metal ion binding	3.42E-08
glycoprotein	4.44E-24	IPR013087:Zinc finger, C2H2-type/integrase, DNA-binding	5.05E-08
glycosylation site:N-linked (GlcNAc...)	2.14E-22	zinc-finger	5.44E-08
GO:0044421~extracellular region part	1.02E-14	GO:0043169~cation binding	9.13E-08
disulfide bond	2.17E-12	domain:KRAB	1.54E-07
disulfide bond	2.61E-12	zinc finger region:C2H2-type 7	1.70E-07
GO:0005576~extracellular region	6.47E-10	zinc	2.42E-07
Secreted	7.57E-10	GO:0043167~ion binding	4.16E-07
GO:0005615~extracellular space	2.98E-06	IPR001909:Krueppel-associated box	4.19E-07
**Enrichment Score: 11.08**		GO:0008270~zinc ion binding	4.72E-07
GO:0031012~extracellular matrix	9.59E-15	IPR007087:Zinc finger, C2H2-type	4.98E-07
GO:0044421~extracellular region part	1.02E-14	zinc finger region:C2H2-type 8	5.34E-07
GO:0005578~proteinaceous extracellular matrix	7.72E-14	zinc finger region:C2H2-type 5	5.86E-07
extracellular matrix	1.87E-10	zinc finger region:C2H2-type 10	7.25E-07
GO:0044420~extracellular matrix part	2.60E-05	zinc finger region:C2H2-type 6	7.70E-07
**Enrichment Score: 9.76**		IPR015880:Zinc finger, C2H2-like	9.93E-07
GO:0001944~vasculature development	1.74E-12	zinc finger region:C2H2-type 9	1.01E-06
GO:0001568~blood vessel development	8.09E-12	zinc finger region:C2H2-type 4	1.10E-06
GO:0048514~blood vessel morphogenesis	2.06E-10	zinc finger region:C2H2-type 2	1.19E-06
GO:0001525~angiogenesis	2.87E-07	zinc finger region:C2H2-type 11	1.38E-06
**Enrichment Score: 7.91**		zinc finger region:C2H2-type 3	1.49E-06
GO:0044459~plasma membrane part	5.45E-13	zinc finger region:C2H2-type 12	2.76E-06
GO:0031226~intrinsic to plasma membrane	1.72E-08	zinc finger region:C2H2-type 1	3.24E-05
GO:0005887~integral to plasma membrane	2.26E-08	SM00349:KRAB	3.66E-05
GO:0005886~plasma membrane	8.75E-08	zinc finger region:C2H2-type 13	2.96E-04

topological domain:Extracellular	1.51E-05	GO:0046914~transition metal ion binding	3.73E-04
**Enrichment Score: 7.51**		SM00355:ZnF_C2H2	3.85E-04
GO:0006928~cell motion	2.92E-11	PIRSF005559:zinc finger protein ZFP-36	4.87E-04
GO:0016477~cell migration	1.36E-07	zinc finger region:C2H2-type 14	0.006676592
GO:0051674~localization of cell	4.78E-07	zinc finger region:C2H2-type 15	0.008464035
GO:0048870~cell motility	4.78E-07	**Enrichment Score: 5.36**	
**Enrichment Score: 7.38**		dna-binding	1.13E-08
cell adhesion	1.82E-10	transcription regulation	1.89E-08
GO:0007155~cell adhesion	5.52E-09	IPR013087:Zinc finger, C2H2-type/integrase, DNA-binding	5.05E-08
GO:0022610~biological adhesion	5.88E-09	Transcription	1.39E-07
GO:0016337~cell-cell adhesion	5.09E-04	IPR007087:Zinc finger, C2H2-type	4.98E-07
**Enrichment Score: 6.60**		IPR015880:Zinc finger, C2H2-like	9.93E-07
GO:0051270~regulation of cell motion	1.93E-09	GO:0045449~regulation of transcription	1.11E-06
GO:0040012~regulation of locomotion	1.84E-08	zinc finger region:C2H2-type 2	1.19E-06
GO:0030334~regulation of cell migration	2.01E-08	GO:0006355~regulation of transcription, DNA-dependent	2.36E-06
GO:0030335~positive regulation of cell migration	5.91E-06	GO:0006350~transcription	4.87E-06
GO:0051272~positive regulation of cell motion	7.71E-06	GO:0051252~regulation of RNA metabolic process	7.69E-06
GO:0040017~positive regulation of locomotion	7.71E-06	GO:0003677~DNA binding	4.48E-05
**Enrichment Score: 6.19**		SM00355:ZnF_C2H2	3.85E-04
GO:0035295~tube development	2.63E-07	GO:0003700~transcription factor activity	7.12E-04
GO:0030324~lung development	6.45E-07	nucleus	0.001656799
GO:0060541~respiratory system development	8.80E-07	GO:0030528~transcription regulator activity	0.007096745
GO:0030323~respiratory tube development	1.13E-06	**Enrichment Score: 4.82**	
**Enrichment Score: 5.06**		GO:0044459~plasma membrane part	5.08E-07
membrane	2.80E-08	GO:0031226~intrinsic to plasma membrane	2.10E-05
transmembrane region	3.62E-07	GO:0005887~integral to plasma membrane	4.57E-05
topological domain:Cytoplasmic	6.12E-07	GO:0005886~plasma membrane	9.93E-05
transmembrane	7.15E-07	**Enrichment Score: 4.19**	
topological domain:Extracellular	1.51E-05	GO:0009952~anterior/posterior pattern formation	1.64E-07
GO:0031224~intrinsic to membrane	0.0044648	GO:0048706~embryonic skeletal system development	2.04E-07
GO:0016021~integral to membrane	0.0125618	GO:0048704~embryonic skeletal system morphogenesis	3.91E-07
**Enrichment Score: 4.54**		GO:0007389~pattern specification process	7.65E-07
GO:0005604~basement membrane	2.07E-05	GO:0003002~regionalization	1.52E-06
GO:0044420~extracellular matrix part	2.60E-05	short sequence motif:Antp-type hexapeptide	2.64E-06
basement membrane	4.20E-05	IPR001827:Homeobox protein, antennapedia type, conserved site	7.55E-06
**Enrichment Score: 4.46**		DNA-binding region:Homeobox	1.25E-05
GO:0030182~neuron differentiation	1.11E-06	GO:0043009~chordate embryonic development	1.55E-05
GO:0000904~cell morphogenesis involved in differentiation	3.84E-06	GO:0009792~embryonic development ending in birth or egg hatching	1.89E-05
GO:0000902~cell morphogenesis	7.53E-06	Homeobox	3.60E-05

GO:0032989~cellular component morphogenesis	7.98E-06	IPR017970:Homeobox, conserved site	3.86E-05
GO:0007411~axon guidance	3.17E-05	IPR001356:Homeobox	4.50E-05
GO:0048812~neuron projection morphogenesis	4.01E-05	GO:0048562~embryonic organ morphogenesis	1.67E-04
GO:0048858~cell projection morphogenesis	5.76E-05	IPR012287:Homeodomain-related	1.70E-04
GO:0007409~axonogenesis	8.32E-05	GO:0048705~skeletal system morphogenesis	1.88E-04
GO:0030030~cell projection organization	8.96E-05	GO:0001501~skeletal system development	2.00E-04
GO:0048666~neuron development	9.16E-05	GO:0003700~transcription factor activity	7.12E-04
GO:0032990~cell part morphogenesis	1.43E-04	SM00389:HOX	9.22E-04
GO:0031175~neuron projection development	1.43E-04	GO:0048598~embryonic morphogenesis	0.002204238
GO:0048667~cell morphogenesis involved in neuron differentiation	3.50E-04	GO:0048568~embryonic organ development	0.003518971
**Enrichment Score: 3.83**		IPR017995:Homeobox protein, antennapedia type	0.005559345
GO:0060348~bone development	9.07E-05	GO:0043565~sequence-specific DNA binding	0.006507222
GO:0001503~ossification	9.56E-05	DNA binding	0.010867953
GO:0001501~skeletal system development	3.68E-04	PIRSF002612:homeotic protein Hox A5/D4	0.011656542
**Enrichment Score: 3.69**		**Enrichment Score: 3.55**	
GO:0000267~cell fraction	1.24E-04	GO:0021545~cranial nerve development	3.85E-05
GO:0005624~membrane fraction	2.15E-04	GO:0021675~nerve development	2.76E-04
GO:0005626~insoluble fraction	3.07E-04	GO:0021602~cranial nerve morphogenesis	0.002097198
**Enrichment Score: 3.57**		**Enrichment Score: 2.95**	
cell junction	4.83E-05	GO:0030182~neuron differentiation	1.61E-06
GO:0045202~synapse	9.02E-05	GO:0048666~neuron development	1.09E-04
GO:0045211~postsynaptic membrane	2.28E-04	GO:0031175~neuron projection development	8.35E-04
postsynaptic cell membrane	4.21E-04	GO:0000902~cell morphogenesis	0.001133352
GO:0044456~synapse part	6.04E-04	GO:0048667~cell morphogenesis involved in neuron differentiation	0.001142704
synapse	0.0013654	GO:0048812~neuron projection morphogenesis	0.001469713
**Enrichment Score: 3.51**		GO:0000904~cell morphogenesis involved in differentiation	0.001924789
IPR000859:CUB	8.43E-06	GO:0048858~cell projection morphogenesis	0.0020366
SM00042:CUB	1.50E-05	GO:0030030~cell projection organization	0.00207289
domain:CUB 2	0.0021594	GO:0032990~cell part morphogenesis	0.003645996
domain:CUB 1	0.0021594	GO:0007409~axonogenesis	0.004438073
domain:CUB	0.0046935	GO:0032989~cellular component morphogenesis	0.006730502
**Enrichment Score: 3.32**		GO:0007411~axon guidance	0.014140307
GO:0009611~response to wounding	4.18E-07	**Enrichment Score: 2.38**	
GO:0006954~inflammatory response	0.0028328	GO:0019887~protein kinase regulator activity	0.00188561
**Enrichment Score: 3.29**		GO:0019207~kinase regulator activity	0.002064811
GO:0016323~basolateral plasma membrane	1.39E-05	GO:0030295~protein kinase activator activity	0.003049091
GO:0070161~anchoring junction	5.16E-05	GO:0030296~protein tyrosine kinase activator activity	0.00757071
GO:0005912~adherens junction	5.19E-05	GO:0019209~kinase activator activity	0.012540543

GO:0005925~focal adhesion	0.0049603	**Enrichment Score: 2.38**	
GO:0005924~cell-substrate adherens junction	0.0072253	IPR015493:Protocadherin beta	3.26E-07
GO:0030055~cell-substrate junction	0.0121063	GO:0007416~synaptogenesis	4.56E-06
**Enrichment Score: 3.15**		GO:0050808~synapse organization	2.26E-05
GO:0051094~positive regulation of developmental process	7.13E-06	IPR013164:Cadherin, N-terminal	0.003489718
GO:0060284~regulation of cell development	4.49E-05	GO:0016339~calcium-dependent cell-cell adhesion	0.006933289
GO:0045597~positive regulation of cell differentiation	7.43E-05	GO:0043062~extracellular structure organization	0.008362278
GO:0050767~regulation of neurogenesis	0.0026131	domain:Cadherin 5	0.020977401
GO:0051960~regulation of nervous system development	0.0040659	GO:0007156~homophilic cell adhesion	0.021137519
GO:0010720~positive regulation of cell development	0.012012	IPR002126:Cadherin	0.026433212
GO:0050769~positive regulation of neurogenesis	0.0285335	GO:0016337~cell-cell adhesion	0.026513559
**Enrichment Score: 3.10**		domain:Cadherin 6	0.02866826
GO:0010033~response to organic substance	8.64E-05	domain:Cadherin 3	0.031947752
GO:0043627~response to estrogen stimulus	7.33E-04	domain:Cadherin 4	0.031947752
GO:0048545~response to steroid hormone stimulus	0.0010084	domain:Cadherin 1	0.037713759
GO:0009719~response to endogenous stimulus	0.0014787	domain:Cadherin 2	0.037713759
GO:0009725~response to hormone stimulus	0.0032735	**Enrichment Score: 2.37**	
**Enrichment Score: 2.92**		GO:0045664~regulation of neuron differentiation	3.89E-06
GO:0030246~carbohydrate binding	1.86E-04	GO:0050767~regulation of neurogenesis	4.61E-06
GO:0001871~pattern binding	9.36E-04	GO:0060284~regulation of cell development	8.13E-06
GO:0030247~polysaccharide binding	9.36E-04	GO:0051960~regulation of nervous system development	5.60E-05
heparin-binding	0.0013813	GO:0045596~negative regulation of cell differentiation	0.001562998
GO:0005539~glycosaminoglycan binding	0.0014483	GO:0010975~regulation of neuron projection development	0.005329877
GO:0008201~heparin binding	0.0085851	GO:0031344~regulation of cell projection organization	0.010157173
Enrichment Score: 2.88		GO:0010769~regulation of cell morphogenesis involved in differentiation	0.010206339
GO:0001763~morphogenesis of a branching structure	8.16E-05	GO:0050770~regulation of axonogenesis	0.013927046
GO:0048754~branching morphogenesis of a tube	8.21E-04	GO:0022604~regulation of cell morphogenesis	0.02876592
GO:0035239~tube morphogenesis	0.002492	**Enrichment Score: 2.35**	
GO:0001569~patterning of blood vessels	0.0165283	cell adhesion	0.001675255
**Enrichment Score: 2.76**		GO:0007155~cell adhesion	0.002817649
domain:NTR	4.86E-04	GO:0022610~biological adhesion	0.002956208
IPR001134:Netrin domain	6.84E-04	GO:0016337~cell-cell adhesion	0.026513559
IPR018933:Netrin module, non-TIMP type	0.0044819	**Enrichment Score: 2.32**	
SM00643:C345C	0.0056124	GO:0019226~transmission of nerve impulse	2.29E-04
**Enrichment Score: 2.75**		GO:0007268~synaptic transmission	0.001047393
GO:0045933~positive regulation of muscle contraction	5.52E-04	GO:0007267~cell-cell signaling	0.003200225
GO:0045987~positive regulation of smooth muscle contraction	0.0013889	**Enrichment Score: 2.21**	
GO:0006937~regulation of muscle contraction	0.0021913	repeat:III	3.58E-05

GO:0006940~regulation of smooth muscle contraction	0.0058329	repeat:IV	3.58E-05
**Enrichment Score: 2.69**		repeat:I	3.58E-05
GO:0040013~negative regulation of locomotion	0.0014972	repeat:II	3.58E-05
GO:0051271~negative regulation of cell motion	0.0020007	hsa04010:MAPK signaling pathway	9.92E-04
GO:0030336~negative regulation of cell migration	0.0027275	GO:0034703~cation channel complex	0.001740068
**Enrichment Score: 2.64**		hsa04930:Type II diabetes mellitus	0.0025295
GO:0031099~regeneration	1.27E-04	GO:0022843~voltage-gated cation channel activity	0.002996565
GO:0042246~tissue regeneration	0.0018619	GO:0005891~voltage-gated calcium channel complex	0.003308564
GO:0040007~growth	0.0043951	hsa05414:Dilated cardiomyopathy	0.005948443
GO:0048589~developmental growth	0.0262396	hsa05412:Arrhythmogenic right ventricular cardiomyopathy (ARVC)	0.007257137
**Enrichment Score: 2.58**		GO:0034704~calcium channel complex	0.008326536
IPR013032:EGF-like region, conserved site	7.87E-07	IPR002077:Voltage-dependent calcium channel, alpha-1 subunit	0.010445678
IPR000742:EGF-like, type 3	6.74E-06	site:Calcium ion selectivity and permeability	0.012878989
egf-like domain	1.01E-05	GO:0005245~voltage-gated calcium channel activity	0.017116854
IPR006210:EGF-like	8.29E-05	GO:0006816~calcium ion transport	0.022324935
IPR006209:EGF	1.03E-04	calcium transport	0.032122609
IPR013111:EGF, extracellular	1.38E-04	hsa05410:Hypertrophic cardiomyopathy (HCM)	0.040719322
domain:EGF-like 5; calcium-binding	1.43E-04	PIRSF005657:voltage-gated calcium channel	0.044867401
domain:EGF-like 3	1.52E-04	region of interest:Binding to the beta subunit	0.047907243
domain:EGF-like 1	1.69E-04	Enrichment Score: 2.15	
IPR000152:EGF-type aspartate/asparagine hydroxylation conserved site	1.95E-04	zinc finger region:C2H2-type 10	7.25E-07
SM00181:EGF	1.95E-04	zinc finger region:C2H2-type 11	1.38E-06
domain:EGF-like 2	2.58E-04	zinc finger region:C2H2-type 12	2.76E-06
domain:EGF-like 4; calcium-binding	2.85E-04	zinc finger region:C2H2-type 13	2.96E-04
domain:EGF-like 5	3.57E-04	zinc finger region:C2H2-type 14	0.006676592
IPR018097:EGF-like calcium-binding, conserved site	5.14E-04	zinc finger region:C2H2-type 15	0.008464035
IPR001881:EGF-like calcium-binding	5.14E-04	**Enrichment Score: 2.02**	
IPR013091:EGF calcium-binding	5.97E-04	GO:0021510~spinal cord development	1.91E-04
domain:EGF-like 3; calcium-binding	7.16E-04	GO:0021515~cell differentiation in spinal cord	0.001833145
SM00179:EGF_CA	9.08E-04	GO:0048663~neuron fate commitment	0.001947932
domain:EGF-like 4	0.001768	GO:0021516~dorsal spinal cord development	0.004164778
domain:EGF-like 7	0.001871	GO:0010001~glial cell differentiation	0.008277744
domain:EGF-like 6	0.0022363	GO:0021780~glial cell fate specification	0.008523566
domain:EGF-like 8	0.0059487	GO:0021778~oligodendrocyte cell fate specification	0.008523566
domain:EGF-like 6; calcium-binding	0.0059487	GO:0021779~oligodendrocyte cell fate commitment	0.008523566
domain:EGF-like 15; calcium-binding	0.00704	GO:0021530~spinal cord oligodendrocyte cell fate specification	0.008523566
domain:EGF-like 14; calcium-binding	0.00704	GO:0021529~spinal cord oligodendrocyte cell differentiation	0.008523566

domain:EGF-like 7; calcium-binding	0.0088403	GO:0048709~oligodendrocyte differentiation	0.028918899
domain:EGF-like 10; calcium-binding	0.0096223	GO:0045165~cell fate commitment	0.041124477
domain:EGF-like 11; calcium-binding	0.012749		
domain:EGF-like 2; calcium-binding	0.0151001		
domain:EGF-like 16; calcium-binding	0.0352581		
domain:EGF-like 13; calcium-binding	0.0352581		
**Enrichment Score: 2.4**			
GO:0044057~regulation of system process	7.29E-05		
GO:0050804~regulation of synaptic transmission	9.15E-04		
GO:0031644~regulation of neurological system process	0.0018372		
GO:0051969~regulation of transmission of nerve impulse	0.002491		
GO:0050806~positive regulation of synaptic transmission	0.0109825		
GO:0051971~positive regulation of transmission of nerve impulse	0.0173372		
GO:0031646~positive regulation of neurological system process	0.0227922		
GO:0048167~regulation of synaptic plasticity	0.0451618		
**Enrichment Score: 2.34**			
GO:0022604~regulation of cell morphogenesis	0.0035548		
cell shape	0.0047177		
GO:0008360~regulation of cell shape	0.005693		
**Enrichment Score: 2.28**			
GO:0009991~response to extracellular stimulus	8.35E-04		
GO:0031667~response to nutrient levels	0.0056747		
GO:0007584~response to nutrient	0.029597		
**Enrichment Score: 2.26**			
GO:0019838~growth factor binding	7.44E-05		
GO:0005520~insulin-like growth factor binding	0.0017469		
domain:IGFBP N-terminal	0.0017495		
IPR000867:Insulin-like growth factor-binding protein, IGFBP	0.0023362		
SM00121:IB	0.0030441		
Growth factor binding	0.0034591		
IPR000716:Thyroglobulin type-1	0.0059739		
SM00211:TY	0.0074568		
IPR017891:Insulin-like growth factor binding protein, N-terminal	0.0085957		
domain:Thyroglobulin type-1	0.0096223		
h_ghrelinPathway:Ghrelin: Regulation of Food Intake and Energy Homeostasis	0.0379799		
**Enrichment Score: 2.26**			
GO:0001667~ameboidal cell migration	0.0012102		
GO:0048762~mesenchymal cell differentiation	0.0037164		
GO:0014031~mesenchymal cell development	0.0037164		
GO:0060485~mesenchyme development	0.0043029		
GO:0014033~neural crest cell differentiation	0.0093014		

GO:0014032~neural crest cell development	0.0093014		
GO:0001755~neural crest cell migration	0.0241813		
**Enrichment Score: 2.16**			
GO:0042981~regulation of apoptosis	3.01E-04		
GO:0043067~regulation of programmed cell death	4.04E-04		
GO:0010941~regulation of cell death	4.63E-04		
GO:0043066~negative regulation of apoptosis	9.33E-04		
GO:0043069~negative regulation of programmed cell death	0.0012356		
GO:0060548~negative regulation of cell death	0.0013082		
GO:0043065~positive regulation of apoptosis	0.0171658		
GO:0006916~anti-apoptosis	0.0180017		
GO:0043068~positive regulation of programmed cell death	0.0190059		
GO:0010942~positive regulation of cell death	0.0205176		
GO:0006917~induction of apoptosis	0.1457275		
GO:0012502~induction of programmed cell death	0.1486095		
GO:0008624~induction of apoptosis by extracellular signals	0.3163828		
**Enrichment Score: 2.15**			
GO:0004714~transmembrane receptor protein tyrosine kinase activity	5.08E-06		
GO:0004713~protein tyrosine kinase activity	0.0023794		
tyrosine-protein kinase	0.0193947		
**Enrichment Score: 2.13**			
GO:0003018~vascular process in circulatory system	9.41E-04		
GO:0008015~blood circulation	0.002469		
GO:0003013~circulatory system process	0.002469		
GO:0050880~regulation of blood vessel size	0.0149121		
GO:0035150~regulation of tube size	0.0149121		
**Enrichment Score: 2.10**			
GO:0034330~cell junction organization	0.0027275		
GO:0007044~cell-substrate junction assembly	0.0054893		
GO:0034329~cell junction assembly	0.0093648		
GO:0048041~focal adhesion formation	0.0265171		

Suppl. Table 6. Upstream regulator analysis between angiogenic (P13) and invasive (P8) tumour cells.

Differentially expressed genes between sorted tumour cells of the angiogenic (P13) versus invasive (P8) phenotype were determined with with the eBayes (Limma) linear model. Cut-offs were set up for FDR<0.01 and abs(FC)>=2. The DEG list was submitted to the Ingenu ty® Pathway Analysis for the analysis of upstream regulators. The regulatory network is considered to be significantly activated if z-score >=2 and inhibited if z-score <=2 (p-value of overlap<0.05).

Upstream Regulator	Activation z-score	p-value of overlap
**Activated**		
TNF	4.68	1.10E-05
NUPR1	4.64	1.95E-05
PDGF BB	4.47	1.43E-03
TGFB1	4.26	2.19E-10
IL6	3.89	1.77E-02
HIF1A	3.66	1.27E-09
MAP2K1/2	3.52	6.37E-05
IL1A	3.45	2.76E-02
ERBB2	3.41	8.42E-06
PI3K (family)	3.34	1.21E-05
IL1B	3.29	3.68E-02
F7	3.25	1.41E-04
STAT3	3.08	4.51E-04
ERK	3.02	1.31E-04
AR	2.99	9.78E-07
NEDD9	2.98	1.96E-09
PRKCD	2.93	2.70E-02
EGF	2.86	4.31E-02
SYVN1	2.83	2.48E-04
OSM	2.81	1.48E-02
ITGB1	2.80	9.39E-03
MCAM	2.59	4.56E-06
PTGS2	2.44	3.15E-03
MYB	2.41	6.65E-03
TWIST2	2.40	2.60E-05
MAPK1	2.39	1.92E-04
P38 MAPK	2.38	2.77E-03
Cg	2.36	5.06E-07
26s Proteasome	2.35	4.64E-03
SMARCA4	2.23	3.79E-09
SCUBE3	2.22	6.32E-04
ETV5	2.22	2.96E-03
ACSL5	2.22	4.79E-02
CTGF	2.18	2.45E-03
EGR1	2.11	3.68E-07
FGF2	2.10	2.75E-05

PKM	2.09	9.52E-04
Jnk	2.05	1.39E-05
TGFBR2	2.04	4.01E-03
TAZ	2.00	7.32E-03
**Inhibited**		
mir-122	-3.61	6.35E-02
SPDEF	-3.58	7.69E-08
FOXA1	-3.29	1.62E-02
PRL	-3.16	8.30E-04
estrogen receptor	-2.99	6.97E-15
miR-29b-3p (and other miRNAs w/seed AGCACCA)	-2.83	1.87E-02
Hdac	-2.74	3.93E-03
COL18A1	-2.65	2.33E-07
WISP2	-2.56	3.85E-04
FBN1	-2.43	2.25E-03
KIAA1524	-2.38	3.85E-02
IgG	-2.35	1.43E-02
IFNL1	-2.28	2.87E-04
miR-124-3p (and other miRNAs w/seed AAGGCAC)	-2.24	3.67E-02
NET1	-2.22	2.03E-04
mir-373	-2.22	1.27E-03
LEP	-2.21	8.91E-02
mir-1	-2.00	9.92E-02

Suppl. Table 7. DAVID analysis for differentially expressed genes (DEGs) between endothelial cells of the angiogenic (P13) tumour and normal brain.

Differentially expressed genes between sorted eGFP^{+}CD31^{+} endothelial cells in P13 xenografts versus endothelial cells in normal mouse brain were determined with the eBayes (Limma) linear model. Cut-off was set up for FDR<0.01. 635 DEGs were divided into upregulated (447) and downregulated genes (188). Separate DEG lists were submitted to the DAVID® database (DAVID 6.7; http://david.abcc.ncifcrf.gov/) for functional enrichment analysis. Main significantly deregulated Gene Ontology (GO) terms, UniProt keywords and features are presented if the enrichment score for the annotation cluster was >2 with terms pvalue<0.05.

**Angiogenic (P13) vs. Normal brain Upregulated**		**Angiogenic (P13) vs. Normal brain Downregulated**	
Term	P Value	Term	P Value
**Enrichment Score: 23.59**		**Enrichment Score: 4.95**	
cell cycle	7.42E-34	glycoprotein	6.14E-11
GO:0007049~cell cycle	6.50E-33	glycosylation site:N-linked (GlcNAc...)	2.43E-09
cell division	3.93E-25	disulfide bond	1.48E-04
GO:0022402~cell cycle process	1.51E-24	disulfide bond	7.56E-04
mitosis	5.60E-24	signal	0.00545914
GO:0000279~M phase	5.53E-23	signal peptide	0.019972351
GO:0022403~cell cycle phase	5.92E-23	**Enrichment Score: 4.42**	
GO:0000278~mitotic cell cycle	8.83E-23	glycoprotein	6.14E-11
GO:0000087~M phase of mitotic cell cycle	1.28E-22	glycosylation site:N-linked (GlcNAc...)	2.43E-09
GO:0051301~cell division	3.37E-22	topological domain:Extracellular	9.88E-06
GO:0007067~mitosis	6.27E-21	membrane	1.21E-05
GO:0000280~nuclear division	6.27E-21	topological domain:Cytoplasmic	1.70E-04
GO:0048285~organelle fission	2.15E-20	GO:0005886~plasma membrane	3.15E-04
**Enrichment Score: 11.5**		transmembrane region	7.64E-04
kinetochore	4.67E-17	**Enrichment Score: 3.87**	
GO:0000775~chromosome, centromeric region	5.32E-16	GO:0043005~neuron projection	1.20E-06
GO:0000793~condensed chromosome	3.52E-14	GO:0030424~axon	1.28E-05
GO:0000779~condensed chromosome, centromeric region	2.63E-13	GO:0042995~cell projection	7.19E-04
GO:0000777~condensed chromosome kinetochore	5.67E-13	GO:0044463~cell projection part	0.02956813
GO:0000776~kinetochore	7.01E-12	**Enrichment Score: 3.22**	
GO:0005694~chromosome	9.64E-12	GO:0050801~ion homeostasis	8.15E-05

centromere	5.11E-11	GO:0006873~cellular ion homeostasis	1.47E-04
GO:0044427~chromosomal part	5.35E-11	GO:0055082~cellular chemical homeostasis	1.82E-04
GO:0007059~chromosome segregation	4.48E-09	GO:0030005~cellular di-, tri-valent inorganic cation homeostasis	2.35E-04
chromosomal protein	2.71E-06	GO:0055080~cation homeostasis	2.97E-04
**Enrichment Score: 8.36**		GO:0019725~cellular homeostasis	3.22E-04
extracellular matrix	4.60E-13	GO:0055066~di-, tri-valent inorganic cation homeostasis	3.97E-04
GO:0031012~extracellular matrix	5.08E-12	GO:0030003~cellular cation homeostasis	5.06E-04
GO:0005578~proteinaceous extracellular matrix	9.63E-12	GO:0048878~chemical homeostasis	5.44E-04
GO:0044420~extracellular matrix part	2.65E-09	**Enrichment Score: 2.54**	
GO:0044421~extracellular region part	2.69E-09	IPR002353:Type II antifreeze protein	1.68E-06
GO:0005604~basement membrane	1.02E-08	GO:0030246~carbohydrate binding	2.63E-06
GO:0005576~extracellular region	9.96E-05	IPR016186:C-type lectin-like	1.53E-04
Secreted	7.65E-04	IPR018378:C-type lectin, conserved site	3.57E-04
**Enrichment Score: 8.09**		GO:0005537~mannose binding	4.67E-04
GO:0005819~spindle	5.56E-13	IPR001304:C-type lectin	6.98E-04
GO:0015630~microtubule cytoskeleton	3.63E-12	Lectin	8.36E-04
GO:0044430~cytoskeletal part	8.71E-10	SM00034:CLECT	8.83E-04
GO:0043228~non-membrane-bounded organelle	1.86E-08	Signal-anchor	0.031688445
GO:0043232~intracellular non-membrane-bounded organelle	1.86E-08	GO:0016044~membrane organization	0.039725883
GO:0005856~cytoskeleton	4.27E-06	Endocytosis	0.052660831
cytoskeleton	8.85E-04	domain:C-type lectin	0.059422095
**Enrichment Score: 4.99**		GO:0016192~vesicle-mediated transport	0.258474476
atp-binding	4.16E-07	GO:0009986~cell surface	0.602502068
GO:0032559~adenyl ribonucleotide binding	7.19E-07	**Enrichment Score: 2.22**	
GO:0001882~nucleoside binding	7.39E-07	GO:0044449~contractile fiber part	2.36E-04
GO:0030554~adenyl nucleotide binding	9.20E-07	GO:0043292~contractile fiber	4.05E-04
GO:0005524~ATP binding	1.03E-06	GO:0030016~myofibril	0.002313657
GO:0001883~purine nucleoside binding	1.24E-06	GO:0007517~muscle organ development	0.005715519
nucleotide phosphate-binding region:ATP	1.39E-06	GO:0030017~sarcomere	0.008861632
nucleotide-binding	2.06E-05	GO:0030018~Z disc	0.011302806
GO:0032555~purine ribonucleotide binding	8.52E-05	GO:0031674~I band	0.01657265
GO:0032553~ribonucleotide binding	8.52E-05	GO:0005198~structural molecule activity	0.821421231
GO:0017076~purine nucleotide binding	9.28E-05	**Enrichment Score: 2.14**	
GO:0000166~nucleotide binding	4.07E-04	IPR001715:Calponin-like actin-binding	0.003300449
binding site:ATP	4.98E-03	IPR003096:SM22/calponin	0.003460241
**Enrichment Score: 4.35**		SM00033:CH	0.003937895
IPR019821:Kinesin, motor region, conserved site	2.75E-06	domain:CH	0.056473043

IPR001752:Kinesin, motor region	3.98E-06	**Enrichment Score: 2.12**	
SM00129:KISc	1.24E-05	GO:0005283~sodium:amino acid symporter activity	3.11E-05
domain:Kinesin-motor	2.04E-05	GO:0005343~organic acid:sodium symporter activity	5.58E-05
motor protein	2.41E-05	GO:0005416~cation:amino acid symporter activity	1.04E-04
GO:0005874~microtubule	4.25E-05	Symport	4.77E-04
GO:0007017~microtubule-based process	7.37E-05	GO:0015370~solute:sodium symporter activity	0.001038492
GO:0003774~motor activity	1.08E-04	GO:0015171~amino acid transmembrane transporter activity	0.001422133
microtubule	1.69E-04	GO:0015293~symporter activity	0.001871331
GO:0003777~microtubule motor activity	3.22E-04	GO:0005275~amine transmembrane transporter activity	0.003336222
GO:0007018~microtubule-based movement	8.99E-04	GO:0005310~dicarboxylic acid transmembrane transporter activity	0.004060049
**Enrichment Score: 3.92**		GO:0006835~dicarboxylic acid transport	0.004373405
cell adhesion	6.63E-07	GO:0015294~solute:cation symporter activity	0.005656587
GO:0007155~cell adhesion	6.41E-05	GO:0046942~carboxylic acid transport	0.013132661
GO:0022610~biological adhesion	6.65E-05	GO:0015849~organic acid transport	0.013584821
GO:0016337~cell-cell adhesion	6.81E-02	PIRSF002444:gamma-aminobutyric acid transporter	0.013692222
**Enrichment Score: 3.88**		IPR000175:Sodium:neurotransmitter symporter	0.013773501
IPR004367:Cyclin, C-terminal	1.16E-05	GO:0005328~neurotransmitter:sodium symporter activity	0.016082552
PIRSF001771:cyclin, A/B/D/E types	3.11E-05	GO:0005326~neurotransmitter transporter activity	0.019306154
IPR014400:Cyclin, A/B/D/E	4.07E-05	GO:0015179~L-amino acid transmembrane transporter activity	0.032461389
PIRSF001771:Cyclin_A_B_D_E	5.09E-05	GO:0006836~neurotransmitter transport	0.037419068
cyclin	9.83E-05	neurotransmitter transport	0.044298891
IPR006671:Cyclin, N-terminal	2.51E-04	transmembrane protein	0.121030659
IPR013763:Cyclin-related	6.63E-04	GO:0006865~amino acid transport	0.138825471
IPR006670:Cyclin	6.63E-04	GO:0031226~intrinsic to plasma membrane	0.198857066
SM00385:CYCLIN	1.35E-03	GO:0015837~amine transport	0.207486269
**Enrichment Score: 3.84**		GO:0005887~integral to plasma membrane	0.294654878
GO:0048514~blood vessel morphogenesis	3.43E-05	**Enrichment Score: 2.09**	
GO:0001568~blood vessel development	3.80E-05	GO:0030247~polysaccharide binding	2.59E-04
GO:0001944~vasculature development	5.20E-05	GO:0001871~pattern binding	2.59E-04
GO:0001525~angiogenesis	5.89E-03	proteoglycan	0.001086963
**Enrichment Score: 3.6**		GO:0044421~extracellular region part	0.005144982
dna replication	6.93E-09	GO:0005539~glycosaminoglycan binding	0.005206773

GO:0006260~DNA replication	4.04E-08	extracellular matrix	0.045053381
GO:0006270~DNA replication initiation	2.07E-06	GO:0005578~proteinaceous extracellular matrix	0.082126703
IPR018525:DNA-dependent ATPase MCM, conserved site	1.41E-05	GO:0031012~extracellular matrix	0.094890049
IPR001208:DNA-dependent ATPase MCM	1.41E-05	GO:0008201~heparin binding	0.194968127
domain:MCM	2.43E-05	**Enrichment Score: 2.0**	
SM00350:MCM	2.62E-05	GO:0008092~cytoskeletal protein binding	7.46E-04
GO:0006261~DNA-dependent DNA replication	1.66E-04	GO:0003779~actin binding	0.023075801
mmu03030:DNA replication	2.89E-04	actin-binding	0.055291356
IPR012340:Nucleic acid-binding, OB-fold	8.25E-03		
GO:0006268~DNA unwinding during replication	2.91E-02		
GO:0032508~DNA duplex unwinding	3.43E-02		
GO:0032392~DNA geometric change	3.99E-02		
IPR003593:ATPase, AAA+ type, core	1.79E-01		
SM00382:AAA	2.74E-01		
**Enrichment Score: 2.88**			
GO:0051651~maintenance of location in cell	3.22E-04		
GO:0051235~maintenance of location	1.22E-03		
GO:0032507~maintenance of protein location in cell	1.59E-03		
GO:0045185~maintenance of protein location	4.75E-03		
**Enrichment Score: 2.8**			
metal ion-binding site:Zinc; catalytic	7.41E-06		
GO:0004222~metalloendopeptidase activity	1.62E-05		
IPR006025:Peptidase M, neutral zinc metallopeptidases, zinc-binding site	1.81E-05		
IPR000884:Thrombospondin, type 1 repeat	3.75E-05		
IPR010294:ADAM-TS Spacer 1	4.17E-05		
IPR013273:Peptidase M12B, ADAM-TS	5.46E-05		
SM00209:TSP1	1.12E-04		
domain:Disintegrin	3.19E-04		
domain:Peptidase M12B	3.69E-04		
cleavage on pair of basic residues	3.78E-04		
IPR018358:Disintegrin, conserved site	4.43E-04		
metalloprotease	5.16E-04		
IPR010909:PLAC	5.17E-04		
GO:0008237~metallopeptidase activity	5.19E-04		
metal ion-binding site:Zinc; in inhibited form	6.70E-04		
IPR002870:Peptidase M12B, propeptide	7.77E-04		

IPR001590:Peptidase M12B, ADAM/reprolysin	9.56E-04		
GO:0004175~endopeptidase activity	1.11E-03		
domain:TSP type-1 1	1.84E-03		
domain:TSP type-1 2	1.84E-03		
domain:PLAC	2.16E-03		
short sequence motif:Cysteine switch	2.65E-03		
domain:TSP type-1 3	4.19E-03		
zymogen	5.98E-03		
region of interest:Spacer	6.70E-03		
GO:0008233~peptidase activity	8.09E-03		
GO:0070011~peptidase activity, acting on L-amino acid peptides	9.23E-03		
IPR001818:Peptidase M10A and M12B, matrixin and adamalysin	9.74E-03		
**Enrichment Score: 2.48**			
GO:0007044~cell-substrate junction assembly	3.06E-04		
GO:0034329~cell junction assembly	1.59E-03		
GO:0034330~cell junction organization	7.73E-03		
GO:0007160~cell-matrix adhesion	7.74E-03		
GO:0031589~cell-substrate adhesion	1.33E-02		

Suppl. Table 8. Comparison of differentially expressed genes (DEGs) in endothelial cells of angiogenic (P13) tumour, intermediate (P3) tumour and normal mouse brain.

Differentially expressed genes between sorted eGFP^{+}CD31^{+} mouse endothelial cells in P13 and P3 xenografts versus endothelial cells in normal mouse brain were determined with the eBayes (Limma) linear model. Cut-off was set up for FDR<0.01. DEGs were divided into upregulated and downregulated genes. Common and unique genes were established by the SUMO software Venn diagram analysis (http://angiogenesis.dkfz.de/oncoexpress/software/).

**Upregulated**				**Downregulated**		
Common	P13 unique		P3 unique	Common	P13 unique	P3 unique
**56**	**391**			**79**	**109**	**57**
2410088K16Rik	1190002H23Rik	Kif20a	Cd72	5033404E19Rik	1700048O20Rik	Adk
Abcg1	1500012F01Rik	Kif22	Clec7a	6330503K22Rik	2610203C20Rik	Ano4
Adamtsl2	2610318N02Rik	Kif23	Cst7	A430107O13Rik	4930506M07Rik	Aoc3
Angpt2	2810417H13Rik	Kif2c	Cxcl13	Abca9	4930594M22Rik	B4galt6
Apln	3110003A17Rik	Kif4	Cxcr4	Abhd3	6720401G13Rik	Bckdhb
Aplnr	4930547N16Rik	Kntc1	Fgfbp1	Acsl6	9530091C08Rik	Bin1
Aprt	4930570D08Rik	Kpna2	Klrb1b	Acta2	Abcd2	Bmp5
Bambi	5730590G19Rik	Kpnb1	Mir92b	Adcy2	Acsbg1	Ccdc66
Ccbp2	6720463M24Rik	Lama4	Olr1	Amigo2	Adcyap1r1	Cd59a
Ccna2	Actl6a	Lamb1	Postn	Appl2	Agmo	Cpm
Ccnb2	Adam12	Lamc1	Tmem154	Bank1	AI464131	Ehd3
Ccnd1	Adam19	Lcp1	Tnf	Bcan	Alas2	Enpep
Cd44	Adamts12	Lgals1		Ccl2	Aoah	Fam107a
Cd93	Adamts2	Lhx6		Ccl24	Arap2	Fam82b
Cdk1	Adamts4	Lipt2		Cd209a	Arhgef6	Fbln5
Ch25h	Adamts7	Lmnb1		Cd209f	Atp13a4	Fmo1
Chst1	Adamts8	LOC1008 61572		Cited4	B230206F22Rik	Fmo2
Cks2	Adamts9	Lpcat1		Cldn10	Bcas1	Fnbp1
Clca5	Adarb1	Lpin3		Cntfr	Cap2	Ggt5
Ctsw	Aldh18a1	Lrp4		Cysltr2	Ccl7	Gm13861
Cxcl10	Angptl4	Lrrc58		Dcn	Ccr6	Gm6712
Dok4	Antxr2	Lrrc59		Dgkb	Cd209b	Gpx8
Dusp2	Anxa2	Mad2l1		Dock10	Cd209g	Gulp1
Ecm1	Apaf1	Man2a1		Ecm2	Cd24a	Hrsp12
Ect2	Arhgap11a	Map3k6		Efhc2	Cdh10	Lactb2
Exoc3l	Arhgap28	Mapk11		F3	Chst8	Lmo7
Fkbp10	Arhgdia	Mastl		Gabrb1	Cmbl	Map2k6
Fscn1	Armcx2	Mcam		Gjb6	Cml2	Mboat2
Gm8817	Arpc1b	Mcm10		Gm106	Cnn1	Mllt3
Htra3	Asap3	Mcm2		Gm10790	Colec12	Mrvi1
Itga4	Asf1b	Mcm3		Gm9946	Crispld1	Myom1

Itga5	Aspm	Mcm4		Gpr37l1	Cryab	Pcdh18
Kcnq1	Atp8b1	Mcm5		Grm3	Cyp27a1	Pla1a
Kctd17	Aurka	Mcm7		Gucy1a3	Ddb2	Pla2g4a
Kit	Aurkb	Mdfi		Gucy1b3	Dmd	Plce1
Lalba	B3gnt3	Med10		Hapln1	Dtna	Pld1
Lipc	Bag2	Megf6		Hey2	Efhd1	Plxnb1
Lxn	Bard1	Mela		Itga8	F630028O10Rik	Polk
Meox1	Birc5	Melk		Itih3	Fgfr3	Ppap2b
Mki67	Bmp1	Mest		Kif5a	Fmn2	Prepl
Nid1	Bmp6	Mgat5		Lama3	Gabbr2	Rasl12
Nid2	Bok	Mis18bp1		Lcat	Gkn3	Rgs7bp
Nlrc3	Btnl9	Mlkl		Mbp	Gm3716	Sdc2
Nos2	Bub1	Mme		Mmd2	Gm3764	Sdpr
Pcdh12	Bub1b	Mmp14		Mrc1	Gm5086	Slc30a10
Prnd	Bzw2	Mmrn1		Mrgprh	Gnao1	Slc38a11
Rhod	C1qtnf1	Mms22l		Myh11	Gpc5	Specc1
Scgb3a1	C1qtnf6	Moxd1		Ndrg2	Gpm6a	Spice1
Serpine1	C230014O12Rik	Mrpl17		Nebl	Gpr160	Tbx18
Slfn9	C330027C09Rik	Mtap		Npy1r	Gpr165	Tmem126b
Spp1	C77370	Mthfd1l		Ntsr2	Gpr183	Tmem47
Tcf19	Cad	Mybl2		Nwd1	Grem2	Tubgcp5
Top2a	Capg	Myh10		Ogn	Gstt1	Zfp141
Trp53i11	Car13	Myo10		P2ry13	Hbb-b1	Zfp442
Tubb6	Casc5	Myo19		Parp8	Hepacam	Zfp62
Ubtd1	Ccdc23	Myo1c		Pdlim3	Hpgd	Zfp930
	Ccl5	Myzap		Peg3	Hpgds	Zkscan3
	Ccnb1	N4bp3		Pf4	Ikzf2	
	Ccne2	Nasp		Plekhb1	Il18	
	Ccnjl	Ncapd2		Pln	Islr	
	Cd109	Ncapg		Psd2	Kif1a	
	Cd276	Ncl		Pygm	Lix1	
	Cd38	Ndc80		Rnd2	LOC100862065	
	Cda	Nek2		Sfxn5	Lrrc49	
	Cdc20	Nln		Slc15a2	Mapk1ip1	
	Cdc25c	Nme1		Slc1a2	Mir505	
	Cdca2	Noa1		Slc26a10	Ms4a7	
	Cdca8	Nop58		Slc39a12	Mustn1	
	Cdh2	Nr5a2		Slc6a1	Nkain4	
	Cdk4	Nrarp		Slc6a11	Nrcam	
	Cdkn2c	Nrp2		Slc7a10	Nrxn1	
	Cdkn3	Nt5dc2		Slc9a9	Olfml1	
	Cdr2l	Nt5e		Sncg	Padi2	
	Cdt1	Nudcd2		Snora20	Pak1	
	Cenpa	Nuf2		Sntb1	Paqr6	

	Cenpe	Nus1		Syne1	Pbld1		
	Cenpf	Nusap1		Tagln	Pcsk1n		
	Cenpk	Ola1		Unc13c	Phkg1		
	Cenpn	Pbk		Vwa3a	Pla2g2d		
	Cenpq	Pcsk6			Plekhn1		
	Cenpt	Pdgfc			Plxdc2		
	Cep55	Pgm1			Ppargc1a		
	Cfb	Pgm5			Ppp1r3c		
	Chac1	Pim3			Ptgds		
	Chaf1b	Plaur			Qpct		
	Chst2	Plekho2			Ralgapa1		
	Chst7	Plk1			Rdh5		
	Ckap2	Plk4			Rragb		
	Ckap2l	Plxnd1			S100b		
	Ckap4	Pole			Scara3		
	Cmtm3	Pole2			Sema3c		
	Cnnm4	Ppa1			Sgip1		
	Cntnap2	Ppat			Siglech		
	Col15a1	Ppic			Slc13a3		
	Col18a1	Ppm1j			Slc13a5		
	Col1a1	Prc1			Slc6a9		
	Col4a1	Prcp			Snx30		
	Col4a2	Prdm1			Sox9		
	Col6a3	Prelid1			Sstr4		
	Coro1c	Prep			Tfrc		
	Creb3l2	Procr			Tlr5		
	Csda	Prr11			Tmem229a		
	Ctgf	Prr5			Tom1l1		
	Cul7	Ptgfrn			Tppp		
	D17H6S56E-5	Pttg1			Tppp3		
	D2Ertd750e	Pvrl2			Tspan7		
	Dbf4	Pxdn			Ttr		
	Dcbld1	Rabggtb			Ttyh1		
	Dck	Racgap1			Wasf3		
	Dctd	Rcan3					
	Ddb1	Rgs9					
	Ddx21	Rhbdl2					
	Depdc1a	Robo1					
	Diap3	Rpl14					
	Dlgap5	Rps6ka2					
	Dmp1	Rrm1					
	Doc2b	Rrm2					
	Dtl	S100a6					
	Dysf	Scn1b					

	E2f1	Sdf2l1				
	E2f3	Sec24d				
	E2f8	Sema3f				
	Eif4ebp1	Serpina3h				
	Emp1	Serpinh1				
	Entpd7	Sesn2				
	Epha2	Sfxn1				
	Esco2	Sgol1				
	Exo1	Sh3bp5				
	Exoc3l2	Sh3gl1				
	Ext1	Shc4				
	F2rl3	Shcbp1				
	F630043A04Rik	Shkbp1				
	Fam102b	Shmt2				
	Fam111a	Slc1a5				
	Fam129a	Slc36a4				
	Fam149a	Slc43a1				
	Fam167b	Slc43a3				
	Fam171a2	Slc7a6				
	Fam198b	Slco2a1				
	Fam38b	Smc2				
	Fam43a	Snd1				
	Fam83d	Snhg1				
	Fbn1	Sox4				
	Fignl1	Spag5				
	Fkbp5	Spc25				
	Flna	Spred3				
	Fn1	Spry4				
	Fosl2	Srgap1				
	Foxm1	Srm				
	Frem2	Srsf2				
	Gabarapl1	Ssr2				
	Gas2l3	St3gal5				
	Gas5	Stc2				
	Gdpd5	Stil				
	Gen1	Svopl				
	Gimap4	Synm				
	Gja1	Tacc3				
	Glra2	Tacr1				
	Gm11567	Taf1d				
	Gm12503	Tfdp1				
	Gm17660	Tfpi2				
	Gm6634	Thbs1				
	Gpr97	Thy1				

	Grp	Timp1				
	Gsto1	Tk1				
	Gtse1	Tmc8				
	H2afx	Tmem120b				
	Hdgf	Tmem22				
	Hist1h1b	Tnc				
	Hist1h2ab	Tnfaip2				
	Hist2h2ab	Tnfrsf10b				
	Hjurp	Tnfrsf23				
	Hmgb2	Tpx2				
	Hmmr	Tram2				
	Hnrnpa0	Trib1				
	Hspg2	Trip13				
	Hyou1	Troap				
	Iars	Tspan18				
	Igf2r	Ttc9				
	Igfbp3	Ttk				
	Igfbp4	Txndc5				
	Ipo5	Txnrd1				
	Iqgap3	Ube2c				
	Irx3	Ube2ql1				
	Itga2	Ubxn8				
	Itga3	Uchl5				
	Itgb3	Uck2				
	Kcna5	Uhrf1				
	Kcne3	Ulbp1				
	Kcnj2	Upp1				
	Kdelr3	Vash1				
	Kif11	Wdhd1				
	Kif15	Wee1				
	Kif18b	Xpnpep1				
		Zfp568				
		Zmat3				
		Zwilch				

Suppl. Table 9. Upstream regulator analysis between endothelial cells of angiogenic tumour (P13) and normal brain.

Differentially expressed genes between sorted endothelial cells of the angiogenic (P13) tumour and normal brain were determined with the eBayes (Limma) linear model. Cut-off was set up for FDR<0.01. The DEG list was submitted to the Ingenuity® Pathway Analysis for the analysis of upstream regulators. The regulatory network is considered to be significantly activated in tumour endothelial cells if z-score >=2 and inhibited if z-score <=2 (pvalue<0.05).

Upstream Regulator	Activation z-score	p-value of overlap
**Activated**		
FOXO1	3.94	4.68E-13
FOXM1	3.89	9.41E-18
TNF	3.77	4.15E-10
ERBB2	3.49	1.31E-31
TGFB1	3.29	2.63E-32
S100A6	2.81	1.63E-06
ERK	2.80	3.16E-03
CTNNB1	2.59	1.63E-06
TRAF2	2.44	1.58E-06
BRD4	2.44	7.79E-03
NFkB (complex)	2.39	4.56E-02
Mek	2.30	3.78E-03
CD24	2.24	2.11E-03
P38 MAPK	2.23	1.60E-05
HIF1A	2.21	6.83E-04
EGFR	2.21	5.69E-04
CSF2	2.14	1.95E-03
RLIM	2.00	1.78E-04
PAF1	2.00	3.55E-02
**Inhibited**		
TP53	-5.55	3.22E-17
NUPR1	-4.96	1.82E-07
estrogen receptor	-3.77	8.43E-05
SPDEF	-3.32	2.77E-07
KDM5B	-3.21	3.99E-08
MGEA5	-3.13	6.46E-05
WISP2	-2.43	9.99E-04
miR-34a-5p (and other miRNAs w/seed	-2.42	3.95E-04

GGCAGUG)		
CDKN1A	-2.27	1.91E-09
ATF3	-2.24	2.85E-06
UXT	-2.23	3.14E-06
miR-483-3p (miRNAs w/seed CACUCCU)	-2.23	1.35E-02
KDM5A	-2.00	5.23E-05
TAB1	-2.00	5.00E-03
DNMT3B	-2.00	1.40E-01

Suppl. Table 10. Comparison of differentially expressed genes in tumour and ECs of the angiogenic phenotype. DEG lists of tumour cells (P13 v. P8 tumour cells, FDR<0.01, abs(FC)>=2) and stromal ECs (P13 v. NB, FDR<0.01, any FC) were compared. Prior to analysis mouse gene IDs were mapped to human gene IDs and unique symbols were extracted. Both DEG lists were split into up and down-regulated genes. Common genes were extracted from Venn diagrams (http://sablab.net). 75 upregulated and 38 downregulated genes were common, 39 genes had an inverse expression levels (not shown).

Common upregulated	Common downregulated
ADAM12	ABHD3
ADAM19	ADCYAP1R1
ADAMTS9	AGMO
ANGPT2	AOAH
ANGPTL4	ARAP2
ANTXR2	ARHGEF6
ANXA2	ATP13A4
APLN	BCAN
ARHGDIA	BCAS1
ARPC1B	CRISPLD1
BMP6	DOCK10
C1QTNF1	FMN2
C1QTNF6	GABBR2
CA13	GNAO1
CAPG	GPR37L1
CD109	GUCY1B3
CD276	HAPLN1
CD44	HEY2
CDKN3	KIAA1161
CHST7	KIF1A
COL4A1	KIF5A
COL4A2	LOC100505572
CSDA	MMD2
CTGF	NDRG2
DCBLD1	NKAIN4
DDX21	NRXN1
EPHA2	PARP8
FAM129A	PPARGC1A
FAM198B	PSD2
FLNA	S100B
FN1	SLC13A5
FOSL2	SLC15A2
FREM2	SNTB1
FSCN1	TOM1L1
GJA1	TPPP
GLRA2	TPPP3

GSTO1	TSPAN7
IGF2R	WASF3
IGFBP3	
IRX3	
ITGA4	
KCNJ2	
KDELR3	
LAMB1	
LAMC1	
LGALS1	
MAN2A1	
MMP14	
MOXD1	
NID1	
NID2	
NOS2	
NRP2	
PDGFC	
PGM5	
PLAUR	
PLXND1	
PPA1	
PPIC	
PRDM1	
PVRL2	
RPS6KA2	
SERPINE1	
SERPINH1	
SHKBP1	
SLCO2A1	
SRM	
SYNM	
THBS1	
TIMP1	
TNC	
TNFAIP2	
TUBB6	
UBTD1	
UBXN8	

Suppl. Table 11. Integrative analysis of protein-protein interactions between tumour and stromal ECs. Cell membrane and extracellular matrix-associated genes upregulated in tumour cells (P13 v. P8 tumour cells, FDR<0.01, FC>=2) and ECs (P13 v. NB, FDR<0.01, FC>1) were compared. Prior to analysis mouse gene IDs were mapped to human gene IDs and unique symbols were extracted. Only direct protein-protein interactions between tumour and EC molecules are shown in the table (tumor specific genes in grey, EC specific genes in green, tumor and EC common genes in red).

Gene A	Gene B		Gene A	Gene B		Gene A	Gene B		Gene A	Gene B
ANXA2	ITGA4		ADAM12	IGFBP5		ANXA2	DYSF		ADM	MME
ANXA2	GJA1		ADAM19	A2M		APLN	APLNR		ADRA1A	BMP1
CD44	IGFBP3		ADAMTS9	LIMS1		CD44	COL1A1		AGT	MME
CD44	ITGA4		ANGPTL4	ITGB1		CD44	DMP1		ANK3	SCN1B
COL4A1	NID1		ANXA2	CD99		CD44	HMMR		ANXA1	DYSF
COL4A1	NID2		ANXA2	ARF6		CD44	SPP1		ARF6	ASAP3
COL4A1	COL4A2		BMP6	BMPER		COL4A1	CD93		BGN	COL1A1
COL4A2	NID2		BMP6	BMPR1A		COL4A2	CD93		BGN	COL18A1
COL4A2	ANTXR2		CD44	HBEGF		CTGF	ITGA5		BMPR1A	BAMBI
COL4A2	NID1		CD44	TGFBR2		CTGF	HSPG2		BOC	CDH2
FN1	THBS1		CD44	SLC7A11		FN1	NT5E		CAV1	PROCR
FN1	IGFBP3		CD44	ITGB1		FN1	ITGA5		CAV1	KCNA5
FN1	ITGA4		CD44	CAV1		FN1	ECM1		CAV3	DYSF
FN1	COL4A1		CD44	ERBB4		FN1	FBN1		CCL2	ACKR2
FN1	COL4A2		CD44	COL1A2		FN1	HSPG2		CD151	PTGFRN
FN1	CTGF		COL4A1	COCH		FN1	ITGA3		CD151	ITGA3
FN1	CD44		COL4A1	DCN		FN1	COL1A1		CDH11	CDH2
IGFBP3	ADAM12		COL4A2	DCN		FN1	CFB		CDON	CDH2
LAMB1	LAMC1		COL4A2	BGN		FN1	ADAMTS4		COCH	COL1A1
LGALS1	GJA1		CTGF	TGFB1		FN1	ITGB3		COL1A2	ITGA2
LGALS1	ITGA4		CTGF	TGFB3		IGFBP3	FBN1		COL1A2	CD93
NID1	LAMC1		CTGF	ERBB4		IGFBP3	COL1A1		COL1A2	COL1A1
NID2	LAMC1		CTGF	NOV		LGALS1	SPP1		COL1A2	ITGB3
PLAUR	IGF2R		CTGF	VEGFA		NID1	ITGB3		COL5A2	BMP1
THBS1	ITGA4		CTGF	LIMS1		NID1	HSPG2		COL8A1	ITGA2
THBS1	COL4A1		EPHA2	EFNA4		NID1	COL1A1		CXCR4	ITGA5
TNC	NID2		EPHA2	EFNA5		NID2	ITGA5		DCN	FBN1
TNC	FN1		EPHA2	CDH5		NID2	ITGB3		DCN	COL1A1
			FN1	SDC2		NID2	HSPG2		DDR2	COL1A1
			FN1	TNFRSF11B		NID2	COL1A1		DPP4	GRP
			FN1	TSHR		NRP2	SEMA3F		DPP4	CCL5
			FN1	ITGA8		SERPINE1	FBN1		DPP4	CXCL10
			FN1	LGALS3BP		THBS1	COL1A1		EDN1	MME
			FN1	LGALS3		THBS1	FBN1		EFNB2	RHBDL2

			FN1	MFAP2		THBS1	ITGB3		F2R	PROCR
			FN1	LTBP1		THBS1	COL18A1		F2RL2	F2RL3
			FN1	NOV		THBS1	ECM1		FBLN5	FBN1
			FN1	MMP2		TNC	ITGA5		FBN2	FBN1
			FN1	GSN					FBN2	ECM1
			FN1	IGFBP5					GRPR	GRP
			FN1	COL1A2					IGF1	IGFBP4
			FN1	COL6A2					IGFBP5	FBN1
			FN1	COL8A1					IGFBP5	SPP1
			FN1	DCN					ITGB1	SPP1
			FN1	C1S					ITGB1	ITGB3
			FN1	ITGB1					ITGB1	ITGA5
			GJA1	NOV					ITGB1	ITGA2
			GJA1	ANXA1					ITGB1	ITGA3
			GJA1	DCN					ITGB1	HSPG2
			GJA1	ATP1A1					ITGB1	COL6A3
			GJA1	CAV1					ITGB1	COL1A1
			IGFBP3	IGF1					JUP	CDH2
			IGFBP3	LTBP1					KCNE4	KCNQ1
			IGFBP3	MMP2					KITLG	KIT
			IGFBP3	CAV1					LOX	FBN1
			ITGA4	ADAM28					LOX	BMP1
			ITGA4	ANXA1					LOX	HSPG2
			ITGA4	RAP2B					LOX	STC2
			ITGA4	SLC2A1					LOXL1	FBN1
			ITGA4	STOM					LOXL1	BMP1
			ITGA4	ITGB1					LTBP1	FBN1
			ITGA4	CXCR4					LTBP2	HSPG2
			KCNJ2	KCNJ16					MFAP2	STC2
			KCNJ2	DLG2					MFAP2	FBN1
			LAMB1	TGFB1					MFAP2	COL18A1
			LAMC1	LAMB2					MFAP2	HSPG2
			LAMC1	LAMA5					MMP2	COL18A1
			LGALS1	LGALS3BP					MMP2	COL1A1
			LGALS1	LGALS3					MMP2	COL6A3
			LGALS1	ITGB1					MSTN	BMP1
			MMP14	LUM					MYD88	CD93
			MMP14	MMP2					NPNT	HSPG2
			MMP14	TIMP3					NRP1	SEMA3F
			MMP14	CAV1					NRP1	ITGA5
			MMP14	COL1A2					PCOLCE	BMP1
			NID1	LGALS3BP					PDGFRA	ITGB3
			NID1	PLXDC1					PDGFRA	KIT
			NID1	MDK					PDGFRB	ITGB3

			NID2	MDK					PTPRE	KCNA5
			NRP2	PLXNA1					PTPRK	CDH2
			NRP2	TGFBR2					PTPRM	CDH2
			NRP2	NRP1					RAP2B	MME
			NRP2	VEGFA					SDC4	CCL5
			PDGFC	PDGFRA					SH2B3	KIT
			PLAUR	LRP1B					SVIL	HMMR
			PLAUR	PDGFRB					TIMP3	ADAMTS4
			PLAUR	ITGB1					TNFRSF1A	SLC1A5
			PLXND1	NRP1					TNFSF10	TNFRSF10A
			PVRL2	USP53					TRIP6	CCL5
			PVRL2	CD226					UTRN	MME
			PVRL2	PVRL3					VEGFA	SEMA3F
			PVRL2	TNFRSF12A						
			SERPINE1	FBLN5						
			SERPINE1	LRP1B						
			SERPINE1	IGFBP5						
			SERPINE1	MFAP2						
			SERPINE1	LOX						
			SERPINH1	STX7						
			SERPINH1	TRIP6						
			THBS1	TNFRSF11B						
			THBS1	TGFB1						
			THBS1	TFPI						
			THBS1	JAG1						
			THBS1	DCN						
			THBS1	IGFBP5						
			THBS1	ITGB1						
			THBS1	MMP2						
			THBS1	BGN						
			THBS1	VEGFA						
			TIMP1	MMP2						

Table 12. Comparison with study by Dieterich et al. (3). DEGs of mouse ECs from angiogenic tumours (P13 v. NB, FDR<0.01, any FC; 599 genes) were compared to previously published list of DEGs in human stroma (Glioblastoma patients v. NB; FDR<0.05, any FC; 95 genes) (3) . Prior to analysis mouse gene IDs were mapped to human gene IDs and unique symbols were extracted. Both DEG lists were split into up and down-regulated genes. Common genes were extracted from Venn diagrams (http://sablab.net).

Common genes deregulated between mouse and human ECs (Glioblastoma v. NB)	
**Common upregulated**	**Common downregulated**
*ANGPT2**	*SLC6A1*
*CD93**	
COL4A1	
COL4A2	
COL6A3	
FN1	
HSPG2	
IGFBP4	
ITGA5	
LAMA4	
LAMB1	
MCAM	
*MMRN1**	
NID1	
NID2	
*NR5A2**	
PXDN	
SERPINH1	
WEE1	

*Genes proposed to be unique to ECs and not present in pericytes by Dieterich et al.

REFERENCES

1. Golebiewska A, Bougnaud S, Stieber D, Brons NH, Vallar L, Hertel F, et al. Side population in human glioblastoma is non-tumorigenic and characterizes brain endothelial cells. Brain : a journal of neurology. 2013;136(Pt 5):1462-75.
2. Verhaak RG, Hoadley KA, Purdom E, Wang V, Qi Y, Wilkerson MD, et al. Integrated genomic analysis identifies clinically relevant subtypes of glioblastoma characterized by abnormalities in PDGFRA, IDH1, EGFR, and NF1. Cancer cell. 2010;17(1):98-110.
3. Dieterich LC, Mellberg S, Langenkamp E, Zhang L, Zieba A, Salomaki H, et al. Transcriptional profiling of human glioblastoma vessels indicates a key role of VEGF-A and TGFbeta2 in vascular abnormalization. The Journal of pathology. 2012;228(3):378-90.
